# Morphology of Nanometric Overlayers Made of Porphyrin-Type Molecules Physisorbed on Cellulose Iβ Crystals and Nanocrystals

**DOI:** 10.1021/acs.jpcb.1c07261

**Published:** 2021-10-12

**Authors:** Agata Fularz, James H. Rice, Pietro Ballone

**Affiliations:** † School of Physics, University College Dublin, Dublin 4, Ireland; ‡ Conway Institute for Biomolecular and Biomedical Research, University College Dublin, Dublin 4, Ireland

## Abstract

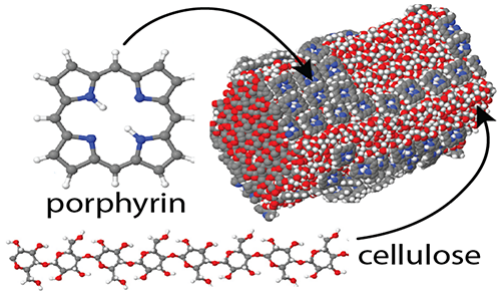

Molecular dynamics simulations based on an atomistic empirical force field have been carried out to investigate structural, thermodynamic, and dynamical properties of adlayers made of porphyrin-type molecules physisorbed on surfaces of cellulose Iβ nanocrystals. The results show that low-index surfaces provide a thermally stable, weakly perturbing support for the deposition of non-hydrogen-bonded organic molecules. At submonolayer coverage, the discoidal porphyrin molecules lay flat on the surface, forming compact 2D clusters with clear elements of ordering. The adlayer grows layer-by-layer for the smallest porphyrin species on compact cellulose surfaces, while forming 3D clusters on a first relatively ordered adlayer (Stranski–Krastanov growth) in all other cases. The adsorption energy exceeds ∼1 eV per molecule, underlying the thermal stability of the adsorbate. Entropy plays a non-negligible role, destabilizing to some extent the adlayer. The in-plane dynamics of the smallest porphyrin species, i.e., porphine, on compact surfaces shows signs of superlubricity, due to the low energy and momentum exchange between the flat admolecule and the equally flat cellulose surface.

## I Introduction

The progressive ban of single-use plastics^1^ by countries and municipalities has spurred the search for natural, recyclable, or degradable alternatives.^2,3^ One of the candidates most likely to play a role in this transition is cellulose, an organic polymer sharing many properties of plastics, but which is natural, easily recyclable, compostable, and biodegradable. Moreover, cellulose is abundant, relatively cheap, and renewable. Last but not least, cellulose has been part of technology and of everyday life for centuries, without manifesting serious environmental problem nor toxic effects.

The structure of cellulose from natural sources is characterized by the alternation of crystalline and glassy domains. Nanocrystalline cellulose, however, can be isolated using a variety of methods,^4^ giving, for instance, cellulose nanofibers (CNFs), made of aligned and ordered polymeric chains, reaching the length of several micrometers. These ordered aggregates can be found in one of several crystal forms.^4^ The focus here is on the Iβ form,^5^ which is present in higher plants and in tunicates. Cellulose nanofibers,^6,7^ in particular, are suitable for advanced applications, providing a stable and biodegradable nanoplatform for drug delivery.^8^ Moreover, CNFs can be functionalized to prepare bactericidal fibers and surfaces,^9^ as well as multifunctional nanoparticles for therapeutics and diagnostics (theranostics).^10^ Other applications in nanotechnology include molecular sensing,^11^ photonics, and organic thin film electronics.^12^ Most of these applications rely on the combination of cellulose with other chemical species, often represented by porphyrin-type molecules,^13^ built around the tetrapyrrole ring of porphyrin, whose central pair of hydrogens is replaced by metal cations lending the sought-after pharmaceutical, catalytic, optical, and electronic properties to the composite material. For instance, upon suitable photoactivation, metal-substituted tetraphenyl porphyrins grafted on the surface of CNFs promote the conversion of ^3^O_2_ to ^1^O_2_, which is cytotoxic but short-lived and can be used to deactivate bacteria^11,14^ and viruses^15^ without significant side effects. As a further example, the inherent tautomerism^16^ of the free base, i.e., non-metal-substituted porphyrins, deposited on surfaces could provide the physical support for cold nanometric storage^17^ and logic devices,^18^ with limited energy requirements and dissipation. Applications of this type require 2D ordered porphyrin adlayers.

In several cases, cellulose functionalization takes place by covalently bonding porphyrins to the surface of CNFs.^13^ In other studies, porphyrins are physisorbed on the cellulose surface. Because of the size and extended π-bonding of porphyrins, the dispersion energy is sufficient to give origin to stable cellulose–porphyrin hybrids, at least in the absence of an organic solvent. Physisorption might have advantages with respect to covalent grafting (chemisorption), because it constrains the resulting structure less and allows the preparation of hybrid systems using relatively simple methods such as the Langmuir–Blodgett approach, vapor deposition, spin-coating, and drop-casting.^19^

The present study is devoted to the computational investigation of the structure, thermodynamics, and dynamics of porphyrin molecules physisorbed on low-index nanocrystalline cellulose surfaces, using molecular dynamics simulations based on an empirical atomistic force field model. The primary aim is to identify the growth mode of the porphyrin adlayer in its early stages of formation, quantify the stability of the porphyrin/cellulose interface, and assess the effect of water contamination on these properties. As already stated, cellulose is represented in its Iβ form. Three free-base, i.e., non-metal-substituted, porphyrin-type species have been considered: (1) the simplest member of the family, i.e., porphine (Porph); (2) a more extended hydrophobic variety, i.e., tetraphenyl porphyrin (TPP); and (3) a cationic species, i.e., tetrakis(*N*-methyl-4-pyridyl) porphine (TMPyP), neutralized by Cl^–^ anions. In what follows, we will refer to any of these three species as *porphyrin molecule*.

In addition to the general reasons of interest discussed in the previous paragraphs, the present series of simulations has been motivated also by the interest in using nanocrystalline cellulose as a support for a variety of experimental techniques, such as, for instance, AFM and vibrational spectroscopy, replacing also in this niche application single-use plastics items. In this context, a moderate adlayer/support interaction is an advantage, and the computational investigation aims at verifying the stability of the cellulose surface upon the deposition of organic species, and the reproducibility of the structure and dynamics of the adlayer molecules, and, possibly, identifying favorable configurations and interactions which could amplify the signal without distorting it.

From a surface science point of view, the general problem we address is that of the growth (for instance by molecular vapor deposition) of a molecular adlayer on the surface of a crystalline substrate. According to a popular surface science textbook,^20^ the adlayer morphology depends primarily on its geometric matching with the surface periodicity of the substrate. The growth will be layer-by-layer (or Frank–van der Merwe) when the matching is good. It will occur by separated islands (or Volmer–Weber)when the geometry of the adlayer and substrate does not match. In the intermediate case of moderate mismatch, islands will grow on one or a few well-ordered but strained adlayers (Stranki–Krastanov growth).

To the best of our knowledge, no previous computational study has been focused on porphyrin/cellulose interfaces, although both CNF surfaces and porphyrin materials have separately been extensively investigated. For instance, ref (4) briefly reviews simulations of nanocrystalline cellulose. Many simulation studies of cellulose crystal surfaces have also been reported,^21,22^ but little quantitative information is provided in the literature beyond the basic features of the structure. Computational and theoretical studies of porphyrins targeted primarily their chemical, electronic, and optical properties,^23^ or their role as prosthetic centers in biosystems. Simulation studies of porphyrin molecules in the soft-matter context, however, have been reported.^24^

From the experimental point of view, many studies have investigated the epitaxial growth of porphyrins on atomistically flat surfaces of noble metals, graphite, salts, oxides, and relatively complex metal–organic framework crystals.^25−29^ The reactivity of porphyrin species on compact metal surfaces has been investigated as well.^30^ The epitaxy of porphyrins on the surface of molecular organic solids, instead, is a relatively new subject.^31^

The results of the present study show that the mobility of the simplest porphyrin, i.e., porphine, on the smoothest cellulose nanocrystalline surfaces is sufficient to ensure layer-by-layer growth. On rougher surfaces, growth turns to Stranski–Krastanov. Because of much lower surface mobility, porphyrin molecules decorated by side groups such as TPP and TMPyP grow according to Stranski–Krastanov even on smooth cellulose surfaces. The addition of water to the interface enhances the mobility of the cationic TMPyP, increasing the ordering of the adlayer. As expected, water does not affect the kinetics and morphology of the hydrophobic adlayers made of Porph and TPP.

## II Model and Method

Molecular dynamics simulations based on an empirical atomistic force field have been carried out using the Gromacs simulation package.^32^ The force field of the Gromos form (version 54A7^33^) has been made by joining and testing force fields for the basic fragments (mono- and disaccharides, and porphyrin-like molecules, parametrized using the ATB website).^34^ The force field has been verified by density functional computations in the PBE approximation,^35^ and carried out using the CPMD package,^36^ again mainly on fragments. Atomic charges and dihedral force constants have been slightly revised as a result of this validation step. The Gromos force field has already been used several times to model cellulose (see, for instance, ref (37)). A more specific cellulose force field has been considered as well,^38^ but eventually, computations have been carried out with Gromos, since this last offers an unbiased description of both cellulose and porphyrins and provides a general framework to extend the present study to further chemical species.

Molecular dynamics has been carried out in the NVT ensemble, since the presence of free surfaces and the large asymmetry of intra- and interchain interactions hamper the application of simple constant pressure algorithms. Care has been taken to remove anisotropic stress components in the samples describing extended surfaces. Constant temperature has been enforced by a Langevin thermostat. Long-range forces have been computed by the PME algorithm.^39^ The analysis of results has been carried out by popular tools such as VMD^40^ and Jmol,^41^ together with simple homemade programs.

The energy of selected crystal surfaces has been computed by a comparison of the potential energy of a bulk and a corresponding slab sample of an equal number of cellulose fibers.^20^ The surface energy computed in this way over a grid of temperatures spaced by 20 K and covering 0 ≤ *T* ≤ 400 K has been interpolated by a Padé polynomial:
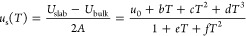
1where *A* is the total surface area. Therefore, by definition, *u*_s_(*T*) is the difference between two primary enthalpy terms, i.e., those of a bulk sample and a slab consisting of the same number of atoms. The harmonic potential energy term (*E*_harm_ = 3*Nk*_B_*T*/2, where *N* is the number of atoms) is the same for the two samples; therefore, *u*_s_(*T*) ∼ *u*_0_ + *αT*^2^ at low *T*, implying *b* = *u*_0_*e* in eq 1. Moreover, the form of the Padé polynomial assumes a linear dependence of *u*_s_ on *T* at high temperature. The interpolation has been used to compute the surface contribution to the specific heat *c*_s_(*T*) (at *P* = 0) and the surface entropy *s*(*T*):
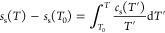
2

Since *u*_s_(*T*) is quadratic at low *T*, *c*_s_ is proportional to *T* in the *T* → 0 limit, and the computation of *s*_s_(*T*) can be carried out setting *T*_0_ = 0 K, and *s*_s_(*T*_0_) = 0. Hence, the surface free energy γ(*T*) can be computed as
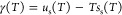
3Moreover, since *s*_s_(*T*) is a difference of entropies, its value can take both positive and negative values without contradiction. Besides computing the entropy and free energy of cellulose surfaces, a similar analysis has been used to quantify the role of entropy in the adsorption of porphyrin molecules.

A significant contribution to the cohesive energy of CNFs is provided by hydrogen bonds, that in our model are identified in terms of geometric parameters only. In cellulose, in particular, each hydroxide oxygen can act at the same time as a proton donor and acceptor. According to our definition, the OH–O triplet is bound by a hydrogen bond if the O–O distance is less than 3.3 Å, and the  angle is ≥140°. Both inter- and intrachain hydrogen-bonding occurs in our samples.

To associate a volume and a density to the molecular adlayer, and to characterize its smoothness or roughness, a geometric surface has been defined by discrete points. For the sake of definiteness, let us consider a cellulose crystal slab and molecular adlayer parallel to the *xy* plane. The location of the upper surface *z*_+_(*x*, *y*) of the sample is defined by a computational AFM as follows. First, we distribute *N*_p_ random points {*x*_*i*_; *y*_*i*_; *i* = 1, ..., *N*_*p*_} on the periodically repeated 2D surface cell. Moreover, we specify the size of atoms, using, for instance, standard tables of van der Waals radii.^42^ Then, for each (*x*_*i*_; *y*_*i*_) point, we lower a spherical tip from well above the sample, moving along *z* in steps *δz* = 0.001 Å. In what follows, the tip has radius *R* = 2 Å, thinner than any realistic AFM tip, but this value can be changed at will, increasing or decreasing the resolution of the map. Then, the surface position *z*_+_(*x*_*i*_; *y*_*i*_) is identified by the first contact of the tip and an atom in the sample. A lower surface *z*_–_(*x*_*i*_; *y*_*i*_) is determined in a similar way for the same set of {*x*_*i*_; *y*_*i*_; *i* = 1, ..., *N*_p_} random points on the surface, starting the tip from well below the slab, moving upward along *z* in the same steps *δz*. We verified that the topography map of the cellulose crystal surfaces reflects the periodicity, symmetry, and structural features that are recognizable in atomistic pictures of the sample, as shown in Figure S1 of the Supporting Information (SI). The histogram of the thickness Δ*z*(*x*_*i*_; *y*_*i*_) = [*z*_+_ – *z*_–_](*x*_*i*_; *y*_*i*_) allows us to compute the volume of the sample (cellulose plus adlayer) as
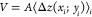
4where *A* is the area of the surface, and ⟨···⟩_*i*_ means average over the {*x*_*i*_; *y*_*i*_; *i* = 1, ..., *N*_p_} points. Subtraction of the cellulose volume determined in the same way upon removing the porphyrin molecules gives the volume of the adlayer. In practice, this approach represents a Monte Carlo determination of the volume.^43^ A random distribution of points has been preferred to avoid any bias toward ordered vs disordered overlayers. The determination of the two surfaces for a single slab configuration takes time on the order of seconds on a single CPU. In principle, the procedure should be repeated for a statistically significant population of sample configurations. However, apart from an initial transient, mobility is low in all samples; hence, only the final configuration has been analyzed in this way.

Smoothness and roughness have been characterized in terms of the height–height correlation function, averaged over the two surfaces limiting each slab:
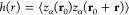
5where α = +, – ; ⟨···⟩ represent the average over the center point **r**_**0**_, over the direction of **r**, and also on α = +, – , when the upper and lower surfaces of the slab are equivalent.

By definition, the (equilibrium) *h*(*r*) correlation function saturates at a constant value with increasing *r* in the case of smooth surfaces, while it diverges logarithmically in the same *r* → *∞* limit for rough surfaces.^20,44^

## III Results

**Figure 1:**
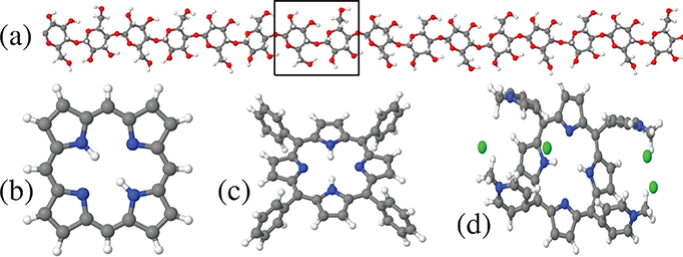
(a) Cellulose chain constituting the polymeric building block in the simulated crystal surfaces and fibrils. Each chain is made of 8 repetitions of the dimeric anhydro-d-glucose unit, identified by the rectangular box. The monoclinic cell of Iβ cellulose encompasses two parallel chains. Gas-phase structure of the porphyrin molecules considered in the present study: (b) porphine, (c) TPP, and (d) TMPyP. Panel (d) shows one of a few low-energy isomers of TMPyP. The tetrapyrrole ring of porphine and TPP is planar, while it is slightly bent in TMPyP. White dots, H; black, C; blue, N; red, O; green, Cl^–^. The different panels are not drawn to scale.

Computations have been carried out on samples prepared in the monoclinic Iβ cellulose form, extensively equilibrated over long simulation times before being exposed to an increasing coverage (θ) of porphyrin molecules. The starting atomistic configuration has been obtained from X-ray and neutron diffraction crystallography data,^45^ manipulated through the online application presented in ref (46). The monoclinic Iβ unit cell, defined by the vectors **a**, **b**, **c** listed in ref (45), is nearly orthorhombic, with an angle γ = 96.5°. Two types of cellulose samples have been considered, the first aiming at describing extended crystalline cellulose surfaces, represented as a slab of ∼4.5 nm thickness. The second one aims at describing nanometric fibers, represented as a cylindrical arrangement of cellulose chains, again of ∼4.5 nm diameter. All simulated systems are periodic in 3D, incorporating a sizable vacuum region to represent 2D surfaces and 1D fibers. In all cases, cellulose chains consisting of 8 dimeric anhydro-d-glucose units (see Figure 1a) were aligned along the longest periodic side (>8 nm) of the simulation box.

Porphyrin-type species considered in our study consist of (i) the archetypal porphyrin molecule, i.e., porphine (C_20_H_14_N_4_, Porph); (ii) the meso tetraphenyl porphyrin (C_44_H_30_N_4_, TPP); and (iii) the meso-tetrakis(*N*-methyl-4-pyridyl)porphine cation (C_44_H_38_N_8_^4+^, TMPyP) neutralized by four Cl^–^ anions, whose gas-phase structure (determined by ab initio and force field models) is shown in Figure 1. Sample sizes ranged from ∼16 000 to ∼32 000 atoms, with simulation times extending up to 200 ns.

### III.A Extended Crystalline Cellulose Surfaces

**Figure 2:**
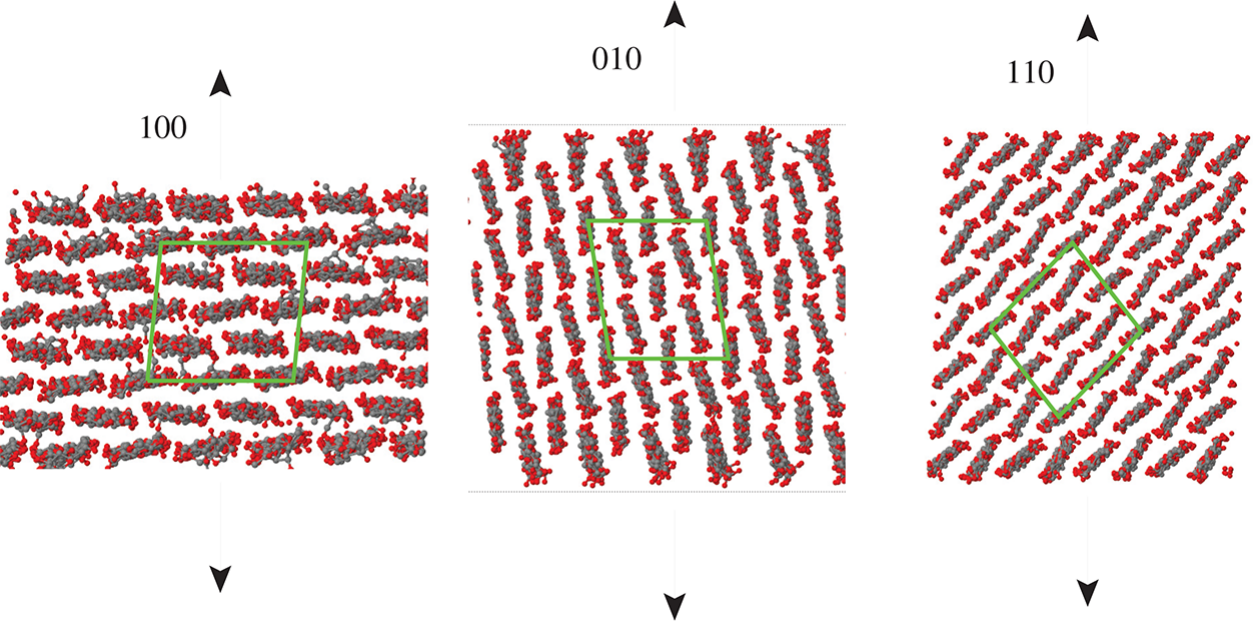
Cross section of the slabs used to represent surfaces, cutting through the covalently bonded cellulose chains. Arrows are perpendicular to the exposed surfaces, and the other two directions are extended through periodic boundary conditions. Because of the low symmetry of the molecular basis of the Iβ unit cell, the upper and lower surface of each slab are not exactly the same. Black dots, C; red dots, O; H atoms not shown. In each panel, the green contour encloses a 2 × 2 replica of the Iβ crystal unit cell.

Paralleling the study of ref (21), three different surfaces have been considered, orthogonal to the (100), (010), and (110) directions. Each cellulose chain may be seen as a ribbon, presenting a relatively flat and hydrophobic top and bottom face, as well as a proton-rich equatorial plane. Because of the low symmetry of the molecular basis of cellulose Iβ, the crystallography of cellulose surfaces is somewhat complicated, and it has become customary to distinguish, for instance, the (100) and (200) surfaces, both orthogonal to the (100) direction, the (010) and (020) surfaces orthogonal to the (010) direction, as well as the (110) and (11̅0) surfaces, which are equivalent in monatomic crystals (see ref (21)). Differences within these pairs of low-index surfaces are relatively minor, and to limit the already large number of simulations, we will not distinguish closely related surfaces, simplifying our notation to (100), (010), (110). To accommodate the lateral periodicity, and to provide sufficient surface area, as well as the adequate separation of the two parallel surfaces, the slabs representing the (100), (010), and (110) surfaces consist of 40, 60, and 64 cellulose chains, respectively. The cross section and the orientation of the slabs used to represent the different surfaces are illustrated in Figure 2. At *T* = 0 K, the area of each surface is *A* = 4215, 3988, 3614 Å^2^ for the (100), (010), (110) cases, respectively. The >4 nm thickness of each slab is sufficient to decouple the upper and lower surfaces. The ≥12 nm periodicity along the direction perpendicular to the slab ensures the separation of opposing periodic images of the surface by nearly 8 nm. Each sample has been first relaxed and then equilibrated at *T* = 300 K for more than 50 ns, reaching the thermodynamic state whose properties are given in Table 1.

**Table 1:** Surface Energy and Surface Free Energy^a^ of the Cellulose Crystal Surfaces Simulated in the Present Study

	(100)	(010)	(110)
*T* = 0 K			
*u*_s_ ≡ γ_f_	71	134	90
*T* = 300 K			
*u*_s_	70	143	106
γ_f_	81	166	129

a Both in mJ/m^2^.

**Figure 3:**
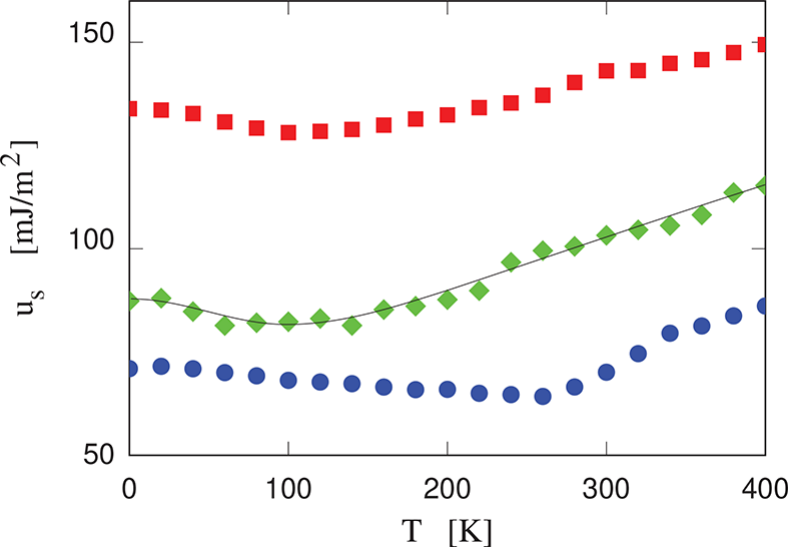
Surface energy of clean crystalline cellulose surfaces as a function of temperature. Blue disks, (100) surface; green diamonds, (110) surface; red squares, (010) surface. Error bars are comparable to the size of the symbols. The thin black line is the Padé interpolation used to compute the entropy contribution to the surface free energy (surface tension) reported in Table 1.

Binding within the slab is ensured by large dispersion energy contributions, and by a fair but not overwhelming number of hydrogen bonds. According to our computations, in the bulk Iβ phase at *T* = 300 K and *P* = 0, there is on average one intrachain hydrogen bond for each pyranose ring, while the number of interchain hydrogen bonds is roughly one-half of the intrachain ones. Long simulations up to 100 ns for the clean surfaces confirm the thermal stability of the Iβ slabs and of their surfaces up to at least 400 K. Thermal decomposition (not described by the force field model) occurs at significantly higher temperatures, exceeding ∼500 K.^47^ The surface energy *u*_s_(*T*) of the (100), (010), and (110) surfaces has been computed over a regular temperature grid spaced by 20 K from *T* = 0 K to *T* = 400 K (see Figure 3). As expected, the (100) surface, smooth and retaining all interchain hydrogen bonds, turns out to be the lowest-energy one, and, for this reason, will be considered the most relevant case, since it will be the most abundant surface in equilibrium nanocrystals. As a comparison, we observe that the *u*_s_ of this crystal surface is comparable to that of liquid water, and this turns out to be in semiquantitative agreement with the results of experimental measurements for microcrystalline cellulose, which, however, refer to a statistical mixture of surfaces, not necessarily representing equilibrium.^48^ The surface energy of the (110) surface is only slightly higher that that of (100), while (010) is significantly higher in energy than the previous two, reflecting the relative smoothness/corrugation of the three surfaces. The temperature-dependent results (see Figure 3) allow us to estimate the surface entropy and then to compute the surface free energy γ(*T*). The temperature dependence of *u*_s_ is moderate and nonmonotonic in all cases; therefore, the role of *s*_s_(*T*) is also moderate but not negligible, as shown by the values for γ(300 K) shown in Table 1. Uncertainties of a few (∼2–3) mJ/m^2^ in *u*_s_ might be due to the challenge of consistently removing strain in the slab and bulk samples at all *T*. The low-*T* portion of the integration in eq 2 tends to amplify the error in *s*_s_(*T*). Because of these uncertainties, *s*_s_(*T*) for the three surfaces is reported in Figure S2 of SI only.

### III.B Porphyrin Molecules on Crystalline Cellulose Surfaces

The evolution of surface properties of cellulose nanocrystals with increasing coverage by porphyrin species has been investigated by progressively adding molecules on each side of the slabs described in the previous subsection. This progressive deposition from the gas phase has been simulated at constant *T* = 300 K. At each stage, the same number of molecules (from 1 to 8) has been added on the opposite sides of the slab, placing them at random in-plane positions, with random orientations, at a distance of about 2 nm above or below the cellulose surface, away from any other molecule that already landed on it. Random deposition continued until reaching 40 molecules per surface in the TPP and TMPyP cases, and 80 molecules per surface in the porphyrin case, corresponding to 2–3 (incomplete) monolayers in each case.

#### III.B.1 One and Two Porphyrin Molecules on the (100) Cellulose Crystal Surface

**Figure 4:**
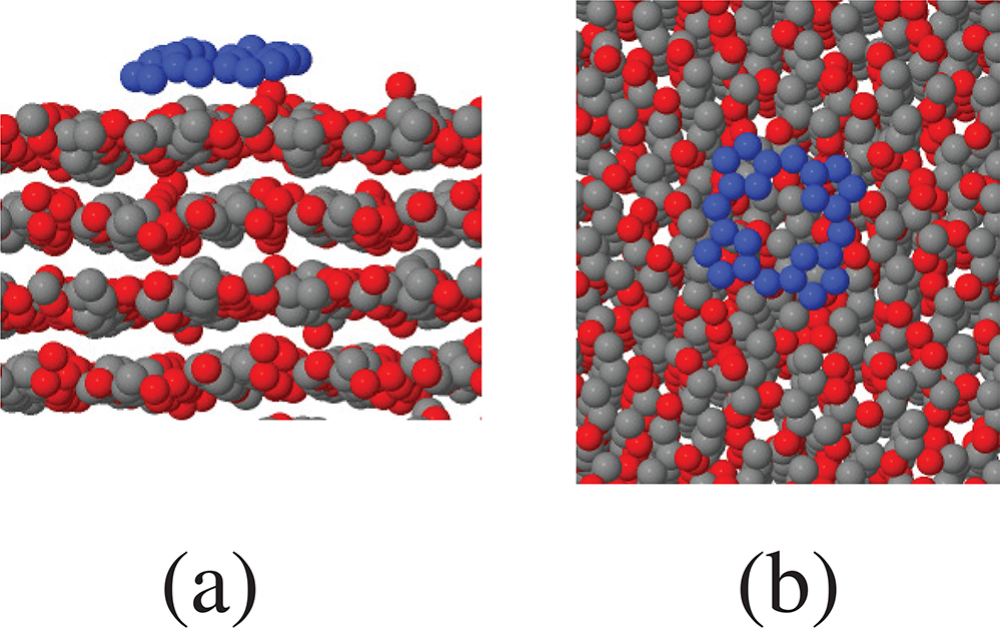
Snapshot of a single porphine molecule on the (100) at *T* = 300 K. (a) Side view. (b) Top view. For the sake of clarity, all porphine atoms are painted blue, and H atoms are not shown.

The simulation with a single porphyrin molecule on each surface provides the baseline to interpret the structure, thermodynamics, and dynamics of adlayers on each surface with increasing coverage, at least up to the monolayer. In the Porph case (see Figure 4), the first two molecules added at random positions and orientation above the two parallel surfaces of the (100) slab have been equilibrated over several nanoseconds. During this time, each molecule landed flat on the (still ordered) surface at *T* = 300 K. At this temperature, the adsorption energy
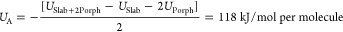
6is high in absolute terms but moderate taking into account the Porph size and the wide (∼100 Å^2^) contact area. In the equation above, the factor of 2 is due to the fact that two Porph molecules have been added, one on each of the two parallel surfaces delimiting the slab. In the convention of eq 6, the attraction between adsorbate and substrate corresponds to positive *U*_A_. Once again, the dependence of *U*_A_ on temperature (see Section S3 in the SI) allows the determination of the entropy contribution to the adsorption free energy *S*_A_. The results show that the adsorption of Porph on the (100) surface of cellulose is driven by a major potential energy contribution (the *U*_*A*_ = 118 kJ/mol per molecule listed above), but entropy plays a non-negligible role, with – *TS*_A_ = 22 kJ/mol per molecule at *T* = 300 K, corresponding to a decrease of excess entropy upon adsorption.

**Figure 5:**
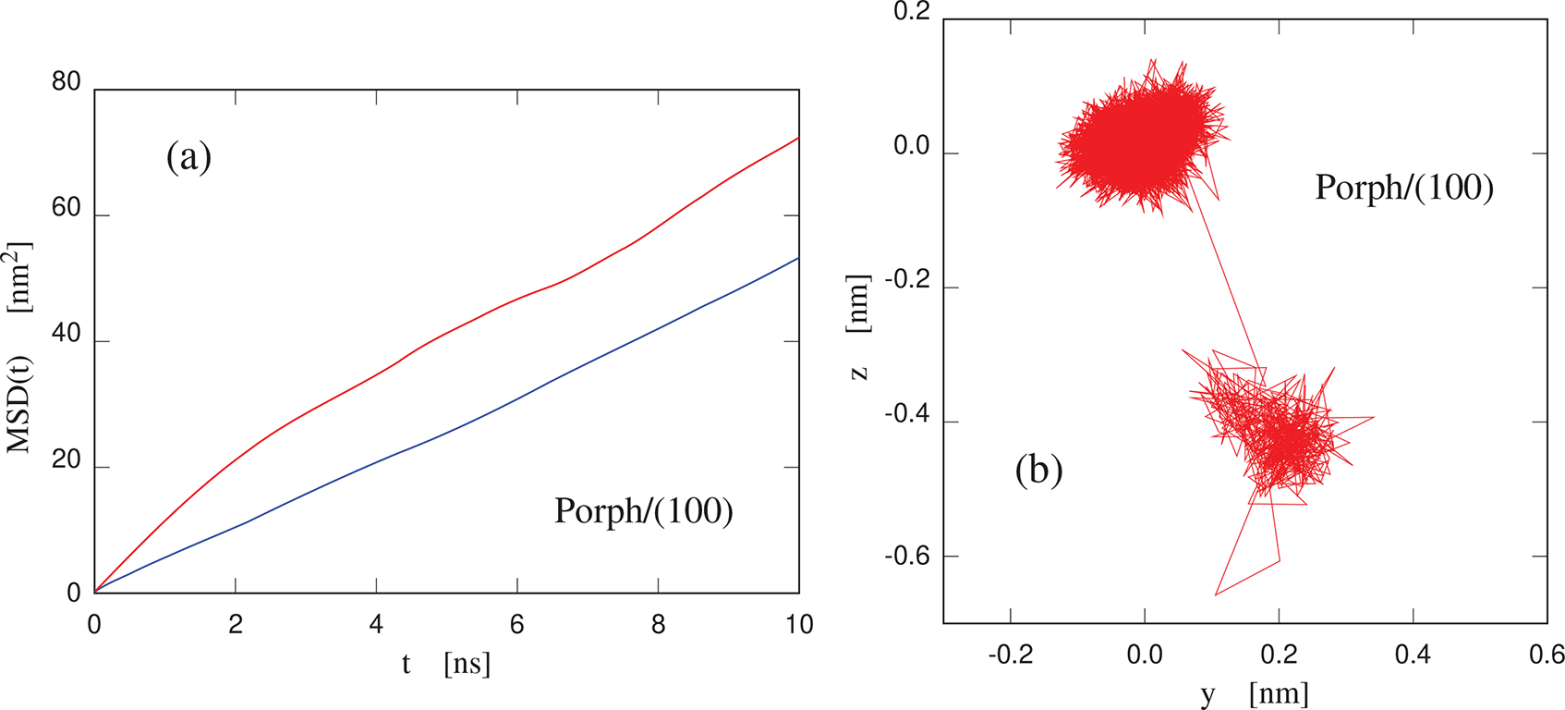
(a) Mean square displacement as a function of time of the center of mass of a single porphine molecule on the (100) surface of cellulose Iβ. Blue line, *T* = 300 K; red line, *T* = 340 K. (b) Trajectory of the same center of mass on the (100) surface. The plot covers 50 ns of MD simulation at *T* = 340 K. The *yz* plane corresponds to the **b** and **c** crystal lattice vectors.

Despite the apparent high affinity of porphine and cellulose, there is no sign of Porph migrating below the surface, perhaps because of kinetic hindrance on the defect-free surface. Once on the (100) surface, the single porphine performs a random walk on its accessible 2D surface space. Analysis of trajectories shows that, if not fully isotropic, diffusion is at least equivalent along the **b** and **c** directions in the surface plane. On the 10 ns scale, the mean square displacement displays a linear dependence on time (see Figure 5), with an estimated 2D diffusion constant *D* = 0.88 ± 0.005 × 10^–5^ cm^2^/s. This is remarkably high for a molecule of the size of porphine (although 2D enhances diffusion), providing a kinetic confirmation of the smoothness of both the (100) surface and the planar porphine molecule. A similar computation at *T* = 340 K gives a diffusion constant *D* = 1.02 ± 0.003 × 10^–5^ cm^2^/s. Although two values of the diffusion constant are certainly not sufficient to verify a genuine Arrhenius behavior, the comparison of the computed values allows us to estimate a diffusion barrier of 1.3 kJ/mol. This is a very low value, which is consistent with the fast surface diffusion of Porph. The nearly dissipation-less diffusion also explains features in the MD trajectory that suggest a clear relation with sophisticated theories of surface diffusion related to Levy flights^49^ and to superdiffusion/superlubricity.^50^ In particular, the high-frequency, short-elongation random motion of the molecule is interspersed with longer jumps over fairly long distances (>5 Å), which at long times (not sufficiently covered by our statistics) modify the simple linear relation of mean square displacement and time characteristic of Brownian motion. Evidence for these jumps is given in Figure 5b and is further documented by the analysis of single trajectories, displaying wide parabolic segments in the plot of the square displacement as a function of time, corresponding to events of nearly rectilinear motion, only weakly damped by friction (see Figure S4 in SI). The explanation of these features is likely to be related to the geometric incommensurability of the porphirine structure and the 2D periodicity of the cellulose crystal surface, preventing the lock-in of porphine into deep energy minima.

All together, these observations show that the dynamics of Porph on the (100) cellulose surface displays many of the characteristic features of fast diffusion and even superdiffusion of molecules on smooth molecular surfaces.^51^

The rotational motion of porphine on the surface is an additional diffusive mode, with a rotational relaxation time of 37 ps (see Figure S5 in SI). Perhaps more importantly, because of the substrate, the Porph orientation is not fully isotropic. This property has been probed by associating a unit vector to each porphine molecule, oriented along the direction joining the two NH groups, and oriented along the diagonal of the (approximatively) square shape of Porph. The distribution of ϕ(*t*) (see histogram in Figure S6 of SI) shows a clear excess at 45°, 135°, 225°, and 315°, implying that the square porphine molecule is preferentially oriented along the crystallographic axes in the plane of the (100) surface.

**Figure 6:**
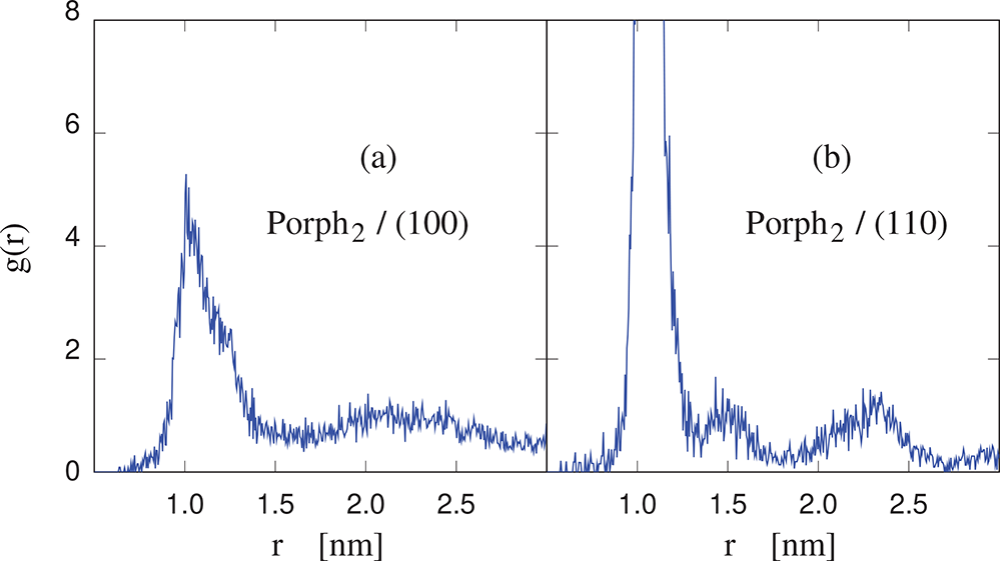
Radial distribution function along the surface (2D) of a pair of porphine (Porph_2_) molecules on (a) the (100) and (b) the (110) surfaces of cellulose Iβ. The radial distance *r* separates the center of mass of the two molecules. At the *r* = 1 nm distance of the main peak of *g*(*r*), the two molecules are in close contact and behave like a dimer.

Simulations of two molecules per surface allow us to assess the pair interaction of two adsorbed molecules, both direct and via the substrate. Simulation snapshots show that Porph molecules invariably lay flat on the surface and retain enough mobility to reach equilibrium within a few nanoseconds. The radial distribution function *g*(*r*) computed for two Porph molecules on the (100) surface (see Figure 6a) shows a first peak at 1 nm, closely corresponding to the side of the square porphine molecule. This peak, already present at the low coverage of 2 molecules per 4215 Å^2^, points to an effective attractive interaction of the two molecules on the surface. On the other hand, the long-range tail of *g*(*r*) shows that the two molecules are not rigidly bound into a dimer but also show some relative translational freedom. These features are consistent with the adsorption energy per molecule computed at *T* = 300 K for the Porph_2_/(100) system, which exceeds that of two Porph_1_/(100) by a relatively modest 8 kJ/mol per molecule, at the limit of our adsorption energy resolution. The Porph–Porph attraction is likely to be a substrate-mediated effect, since the H-saturated boundary of porphine appears to be rather inert. We point out that the observation of dimers made of aligned and closely spaced porphine molecules on the surface might have a different interpretation, and the truth is likely to be a combination of the two pictures. The tendency to form closely connected pairs, in fact, could be an excluded volume (excluded area) effect, due to entropy. In a similar way, the radial distribution function of hard spheres (disks) has its highest peak at contact, despite the absence of any attractive interaction. Some role for a substrate-mediated attractive interaction, however, is confirmed by the computed (8 kJ/mol per molecule) excess adsorption energy, and by the comparison with the radial distribution function computed for the same two Porph molecules on the (110) surface, displayed in Figure 6b, and showing sizable differences from the (100) case.

The adsorption of TPP and TMPyP on the (100) surface in the low-coverage limit shows similarities and differences with the Porph case. On one hand, adding one and two TPP or TMPyP molecules at random positions (and orientations) above and below the (100) slab quickly gives origin to configurations similar to those of Porph/(100), with the molecules laying flat on the surface, leaving the cellulose surface structure only slightly perturbed by the adsorbate molecule. The tetrapyrrole core of Porph, TPP, and TMPyP adsorbed on (100) is planar. Moreover, to a good approximation, the peripheral rings of TPP and TMPyP are nearly orthogonal to the macrocycle plane, with an average tilt of ∼20°. The adsorption energy of Porph, TPP, and TMPyP on (100) (see Table 2) grows in parallel with the increasing molecular size, both at *T* = 0 K and at *T* = 300 K. On the other hand, the surface mobility of TPP on (100) is 2 orders of magnitude lower than that of Porph, and the mobility of TMPyP is even much lower, preventing the reliable computation of the diffusion coefficient. The low mobility, moreover, makes the discussion of properties such as rotational diffusion and preferential orientation with respect to the substrate uncertain.

**Table 2:** Adsorption Energies *E*_A_^a^ of Isolated Porphyrin Molecules on the (100), (010), and (110) Surfaces of Crystalline Iβ Cellulose^b^

surface	Porph	TPP	TMPyP
*T* = 0 K			
(100)	124	203	389
(010)	184	165	524
(110)	210	134	379
*T* = 300 K			
(100)	127	181	398
(010)	118	164	450
(110)	111	106	416

a In kJ/mol per molecule.

b Error bars are of the order of 5 kJ/mol per molecule.

#### III.B.2 One and Two Porphyrin Molecules on the (010) and (110) Cellulose Crystal Surfaces

The clean (110) slab has surface energy not much higher than that of (100), and its structure appears to be fairly compact and smooth. The major difference with respect to (100) is that a sizable number of polar OH groups are exposed on the surface, providing opportunities for H-bonding. The porphyrin molecules considered in the present study, however, do not participate in H-bonding; therefore, their adsorption properties on (110) are expected to be similar to those found for the (100) case.

In the low-coverage limit, the process of landing and thermalizing porphyrin molecules on the surface following their random distribution above and below the slab is in fact similar in the (100) and (110) cases, achieving apparent equilibration within a few nanoseconds. Especially, at low *T* (see the *T* = 0 K values of *E*_A_ in Table 2), adsorption energies computed for Porph, TPP, and TMPyP on (110) (one molecule per surface) are not easily rationalized in terms of molecular size or of the corresponding (100) values, pointing to the role of quantitative matching of surface periodicity and structural details for each of the adsorbed molecules. Mobility on (110) is lower but comparable to that on (100) for the single Porph molecule, too low to make a quantitative comparison in the case of TPP and TMPyP.

**Figure 7:**
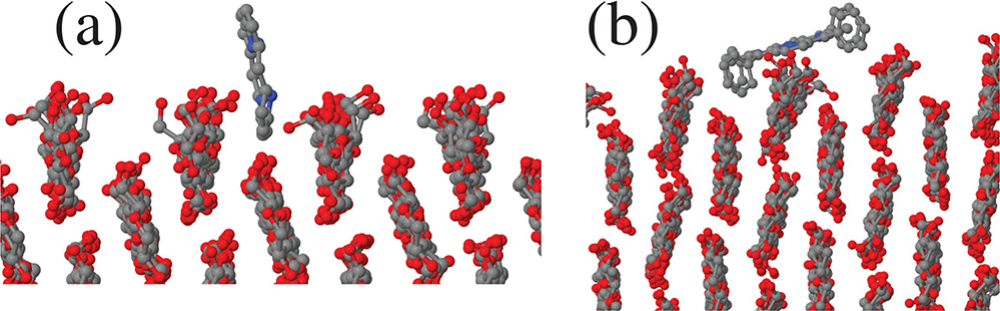
Snapshot of porphyrin molecules deposited on the (010) surface of cellulose Iβ at low coverage. (a) Porphine (Porph). (b) TPP.

The surface of the (010) slab is much less compact than in the previous cases, presenting deep narrow grooves, as reflected in a surface energy significantly higher than for (100) and (110). From the structural point of view, therefore, the adsorption of flat porphyrin-like molecules is less regular than in the previous cases; since newly added molecules end up in a variety of different configurations (see Figure 7), their mobility is negligible, and attaining thermal equilibrium is far from certain on the simulation time scale. Especially at low temperature, molecules may end up being partially incorporated into the grooves, accessing more contact area and gaining more adsorption energy than on (100). Even more than in the (110) case, the low-temperature adsorption energies of Porph, TPP, and TMPyP on (010) do not simply parallel the molecular size and the (100) values but show values that reflect the matching of the surface periodicity with molecular features. At a higher temperature, the role of deep and narrow valleys in the potential energy surface of molecules on (010) is decreased by entropy, and adsorption energies are somewhat more predictable (see the *T* = 300 K values of *E*_A_ in Table 2).

As a consequence of the rough interface, the mobility of porphyrin molecules on the (010) surface is particularly low. Interestingly, the anisotropy of the surface is reflected into the anisotropy of the mean square displacements, that in all cases turn out to be higher along the *z* direction parallel to the grooves (see Figure S7 in the SI).

#### III.B.3 Adlayer Evolution with Increasing Coverage

**Figure 8:**
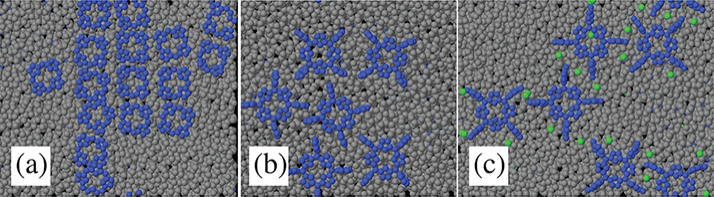
Snapshot of porphyrin molecules (Porph, panel a; TPP, panel b; TMPyP, panel c) adsorbed on the (100) surface of cellulose Iβ at low coverage. All samples are well-equilibrated (simulation time >50 ns) at *T* = 300 K. In all cases, the horizontal axis is parallel to the direction of cellulose chains, showing that the orientation of the molecules on the surface is biased by the underlying crystal structure.

The progressive addition of molecules on the (100) surface at coverage θ < 1 monolayer (ML) reveals a remarkable tendency to ordering in the initial growth of Porph, TPP, and TMPyP adlayers. In all these cases, molecules lay nearly flat (with a slight systematic tilt of the macrocycle plane) on the cellulose surface, confined by sizable dispersion interactions, weakly bound among themselves by lateral attractive forces, giving origin to finite 2D clusters characterized by a very recognizable simple geometry (see Figure 8), that, given time and favorable boundary conditions, could grow into periodic structures. Experimental studies^25,26,31^ of the structure of porphyrin molecules (mainly TPP) on HOPG or on smooth metal and organic crystal surfaces show a similar tendency toward regular epitaxial monolayers, whose periodic structure is based on either square or hexagonal unit cells. Our simulations for porphyrins on the (100) and (110) surfaces reveal the same square and hexagonal motifs. In this respect, the cellulose substrate does not seem to be very selective, with both square and hexagonal clusters being present, sometimes even in the same adlayer at submonolayer coverage, with, however, a quantitative prevalence of hexagonal clusters.

**Figure 9:**
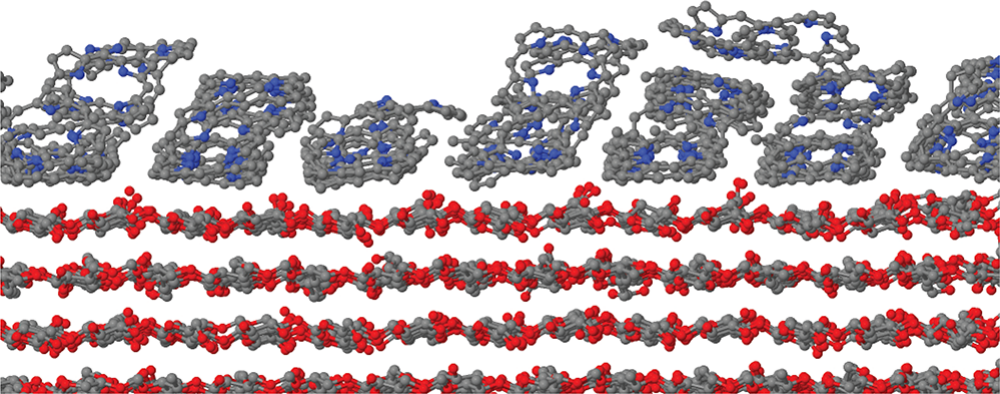
Snapshot of Porph molecules adsorbed on the (100) surface of cellulose Iβ at coverage θ > 1. In the vapor deposition process we simulated, growth takes place layer-by-layer (or Frank–van der Merwe, see ref (20)).

In the case of the porphine molecule (molecular area ∼100 Å^2^), the formation of the second adlayer on the (100) simulated surface (area 4215 Å^2^) starts at a coverage slightly above 24 molecules, when the first layer is only 60% complete. Up to the highest coverage considered in the simulation, the residual Porph mobility, especially for the top-most incomplete layer, is sufficient to reach a fair degree of ordering. Porphine molecules, in particular, tend to arrange on top of each other, although because of the slight tilt, the axis of the growing pillar is not orthogonal to the cellulose surface (see Figure 9).

**Figure 10:**
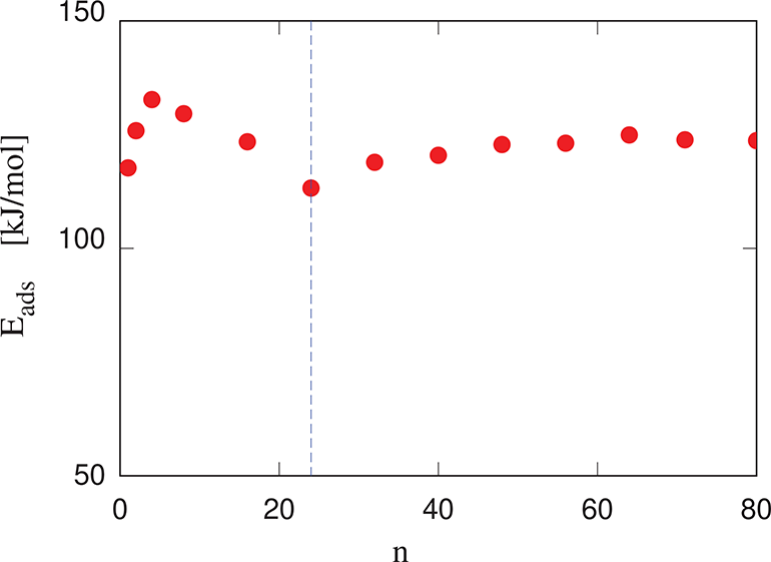
Adsorption energy *E*_A_ of porphine (Porph) molecules on the (100) surface of cellulose as a function of coverage, measured by the number of molecules deposited on each surface (4215 Å^2^) of a planar slab. The energy is in kJ/mol per molecule. The vertical dashed line marks the beginning of the second overlayer formation.

The adsorption energy *E*_ads_ for Porph on (100) shows a clear anomaly at the coverage (24 molecules per 4215 Å^2^ surface area) at which the second adlayer starts to form (see Figure 10). The anomaly, however, is relatively weak. Apparently, from the point of view of the dispersion energies driving adsorption, the binding of Porph to the flat (100) cellulose surface, or to a porphine already on the surface, is nearly equivalent, since cellulose and the adlayer are of comparable average electron density and polarizability. Computer adsorption energies at *T* = 300 K are compatible with the very few data from experiments,^52^ showing that the sublimation enthalpy of TPP at the same temperature is about 150 kJ/mol. In this semiquantitative comparison, we assume that sublimation from a TPP solid is not too different from sublimation from a cellulose adlayer.

The very early stages of growth on (100) are similar for Porph, TPP, and TMPyP. Soon, however, the much lower mobility of TPP and TMPyP changes their growth from layer-by-layer to 3D clusters on top of the first fairly regular adlayer, even for the smooth (100) surface. This might also be due to the short time scale of the simulation, but it is likely to be true also on macroscopic time scales at coverage beyond the monolayer, since the mobility of TPP and TMPyP decreases even further with increasing thickness of the molecular adlayer. Experiments show a similar Stranski–Krastanov growth for TPP even on the atomistically smooth surface of highly oriented pyrolytic graphite.^53^

**Figure 11:**
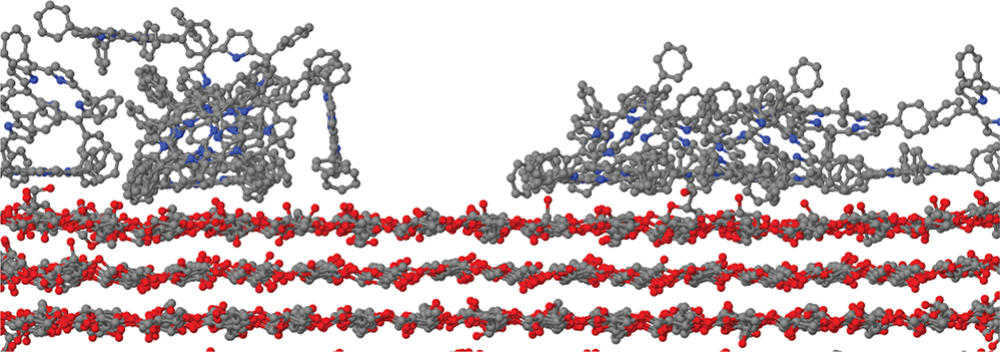
Snapshot of TPP molecules adsorbed on the (100) surface of cellulose Iβ at coverage θ ∼ 1. In the vapor deposition process we simulated, growth takes place by disordered 3D clusters on a relatively ordered first monolayer (Stranski–Krastanov).

The same picture is observed for growth on the (110) surface around and above ML coverage. The fair mobility of Porph on (110) gives origin to ordered layer-by-layer growth (see Figure S8 in SI), while TPP and TMPyP form 3D disordered clusters for θ > 0.5 ML (see Figure 11), growing on a first incomplete but relatively ordered flat layer on the surface.

Again, low surface mobility determines the 3D cluster growth of Porph, TPP, and TMPyP on the (010) surface at all coverages such that the number of admolecules in the simulation exceeds the very few (see Figure S9 in SI).

**Figure 12:**
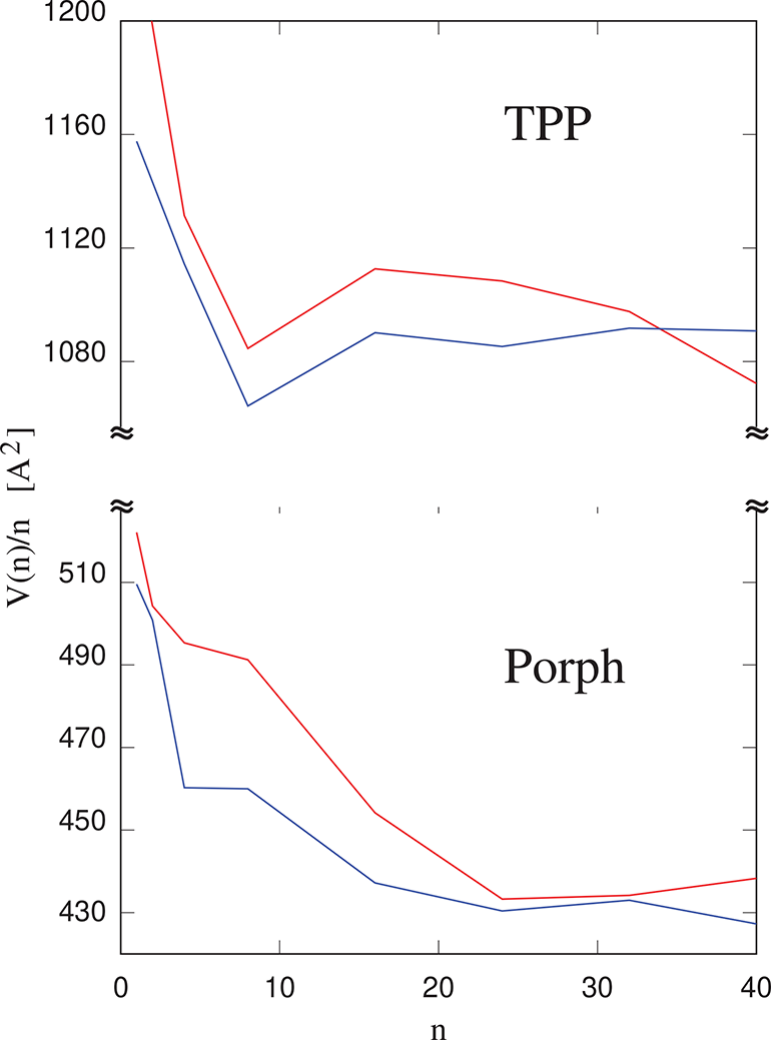
Volume per adsorbate molecule as a function of coverage, measured by the number *n* molecules deposited on each surface of the planar slabs described in Section IIIa. The red curves refer to molecules on (100), and the blue curves refer to molecules on (110).

To quantify the degree of self-organization of molecules on the different surfaces, the adlayer volume (and thus density) has been determined by the virtual AFM approach described in Section II. This determination proceeds in two steps, first computing the volume of the entire sample, and then the volume of cellulose alone, upon removing the adlayer at fixed cellulose atom positions. The approach somewhat overestimates the volume, since this accounts also for the nonvanishing size of the probe particle (as a comparison, the volumes of TPP in the triclinic^54^ and tetragonal^55^ crystal are 802 and 799 Å^3^, respectively), but it correctly reflects the trends as a function of coverage and of surface type. First of all, the results show that the volume of the underlying cellulose slab depends only weakly and nonsystematically on coverage. The adlayer volume per molecule, instead, shows a dependence on coverage that, not surprisingly, is different for layer-by-layer growth and for 3D clusters growth. For instance, the volume per Porph molecule on (100), representing the paradigmatic layer-by-layer growth, at first decreases rapidly with increasing coverage, and then it saturates at θ > 1 coverage (see Figure 12). The volume per molecule of TPP on (100) also decreases rapidly at first but then increases again when the growth turns to 3D (rather disordered) clusters. Since, in the present model, intermolecular interactions are pairwise additive, the initial rapid decrease of the volume per molecule cannot be attributed to cooperative binding of molecules to the surface, as confirmed also by the fact that the cohesive energy *U*_A_ does not depend much on coverage. As suggested also by snapshots, the effect seems to be due to the formation of compact 2D clusters, which reduces the overall volume of the adsorbate. A similar behavior and similar trends are observed for Porph and TPP on the (110) surface. The initial shrinking of the adlayer volume per molecule is not observed for TMPyP on all surfaces, perhaps because the system grows via 3D clusters starting from relatively low coverage. On (010), all three porphyrin species grow 3D from the beginning, and the volume per molecule does not show a systematic trend as a function of coverage, apart from a slow overall growth with increasing coverage.

The data on the adlayer height as a function of position allow the computation of the height–height correlation function. Unfortunately, the system size and the limited range of coverage accessible to MD simulation seem to greatly affect the results. Not surprisingly, with increasing *r*, *h*(*r*) tends to a constant for Porph on (100) and (110), confirming the surface smoothness of the adlayer growing layer-by-layer. However, also, the systems growing by 3D clusters display an *h*(*r*) autocorrelation function that saturates at the longest distances accessible in the simulated samples, although at a significantly higher value than in the previous cases (see Figure S10 in SI). In other words, the logarithmic divergence of *h*(*r*) characterizing rough surfaces is not observed even for adlayers growing by 3D clusters. Much larger simulations would be needed to quantify roughness vs smoothness, but these would be better carried out using a coarse-grained approach.

To verify the stability of the adlayer structures, samples at the highest coverage on each surface have been progressively heated to *T* = 360 K during 60 ns, and (linearly) annealed back to *T* = 300 K during another 60 ns. No change is visible in the simulation snapshots, nor can be detected by our analyses, suggesting that the picture provided in this section remains valid over a somewhat longer time scale than probed directly by our (relatively short) simulation.

Last but not least, we comment on the electrostatic signature of the different overlayers. As expected, the only relevant case is represented by the TMPyP overlayer, since clean cellulose slabs and Porph or TPP overlayers have a negligible dipole moment. The TMPyP overlayers, instead, have a nonvanishing dipole moment because of the preferential adsorption of Cl^–^ at the cellulose surface, due to its electrostatic attraction to the positive end (proton) of the OH cellulose groups, and to the relatively low size of the Cl^–^ anion. In other terms, the positive and negative charge distributions in the adlayer are slightly shifted with respect to each other, with the negative part closer to cellulose. As a result, the TMPyP adlayer on each side of the slab presents an average dipole perpendicular to the interface, pointing to the vacuum side. The effect might be amplified by the low molecular mobility in the adlayer, since in a liquid system the Cl^–^ surface charge would be screened over a shorter distance. Therefore, the entire adlayer behaves as the dielectric inside of a capacitor, whose plates are represented by the cellulose–adlayer interface, and by the adlayer-free surface. Because of the glassy state of the adlayer, the equilibrium value of the dipole is somewhat uncertain. Nevertheless, the average dipole moment per unit surface seems to grow nearly linearly with coverage, and at the maximum coverage of our simulations (2–3 monolayers), it reaches ∼1 D/nm^2^ for the (100) slab, ∼1.4 D/nm^2^ for (110), and ∼1.7 D/nm^2^ for (010), with an uncertain error of the order of 20% (estimated by fluctuations). The electric field arising inside the capacitor breaks the symmetry of the environment even relatively far from the interface and could affect vibrational and optical spectroscopic properties. Extended polar surfaces are intrinsically unstable, but the micrometric length and nanometric diameter of the CNF might be compatible with a sizable surface dipole.

### III.C Effect of Water Contamination

Potential applications of porphyrin-based adlayers on nanocrystalline cellulose surfaces^9,11^ will presumably take place in an open environment, with water present as a primary component or as a contaminant. To assess the effect of water on the structure and dynamics of these systems, simulations have been carried out on selected adsorbate/cellulose samples upon addition of 1000 water molecules. To provide a benchmark, samples made of cellulose and water only have been simulated for ∼100 ns at *T* = 300 K. In all cases, water does not enter the cellulose slab but partially wets the cellulose surface, forming confined drops (see Figure S11 of SI) with a contact angle between 45% and 60%. Applications of macroscopic thermodynamic relations show that the water/cellulose interfacial free energy is ∼50% of the (quantitatively similar) surface free energy of cellulose and water.

Since both Porph and TPP interact only weakly with water, simulations with water and porphyrin adlayers have been focused on the TMPyP samples at coverage of 40 molecules on each of the two surfaces limiting the (100), (110), and (010) slabs. At this coverage, the 1000 water molecules represent 18 wt % of the total (water + TMPyP) adlayer mass.

During simulations, water is added to the already deposited TMPyP adlayer from the vacuum side, starting from random positions and orientations. The first observation is that, in all cases, water enters the adlayer permeating it in subnanosecond times, possibly because of crevices in the disordered structure. On the other hand, water remains outside the cellulose slab. Moreover, despite its incorporation into a complex ionic solid, water retains a fair mobility (∼ 10^–6^ cm^2^/s at *T* = 300 K) that persists over time. Water seems to increase the mobility also of TMPyP and Cl^–^, but this remains too low to be measured with confidence. The general increase of mobility, however, might be inferred from the fact that on all surfaces the structure of the wet adlayer appears somewhat more ordered than for dry samples. The ordering effect is greatly amplified by the heating (60 ns)/cooling (60 ns) cycle from *T* = 300 K to *T* = 360 K and back, already applied with little effect to selected dry samples. In the case of wet samples, the increase of ordering is apparent already from snapshots, emphasizing the role of water as molecular lubricant (see Figure S12 in SI). Ordering, in particular, corresponds to the fact that there is at least one full layer of TMPyP molecules parallel to the cellulose surface, again giving Stranski–Krastanov growth.^20^ Moreover, TMPyP molecules in further layers also show a tendency to orient themselves parallel to the cellulose surface, although their structure remains fairly disordered, also with large deviations in orientation. More importantly, the significant effect of a 120 ns thermal cycle suggests that, for wet TMPyP overlayers, ordered structures could be obtained already on microsecond time scales, at least in the first few adlayers close to the surface of nanocrystalline cellulose.

The incorporation of water into hidden cavities and the annihilation of defects due to enhanced mobility are emphasized by the volume nonadditivity of adlayer and water. In more detail, the 1000 water molecules occupy, at normal conditions, ∼30 000 Å^3^. Their addition to the TMPyP adlayer on (100), however, increases the system volume by only ∼8000 Å^3^, as we verified by virtual AFM measurements.

### III.D Nanocrystalline Cellulose Fiber

**Figure 13:**
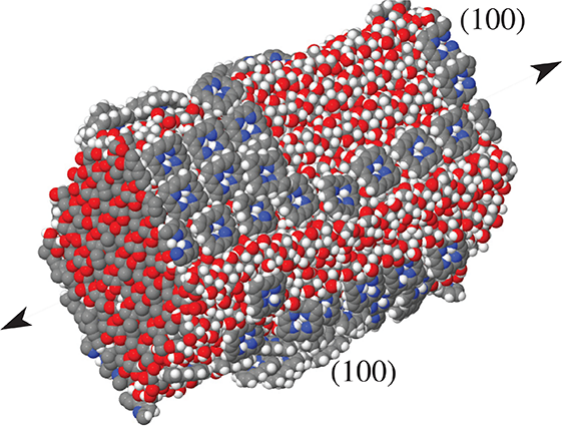
Snapshot of the nanocrystalline fiber simulated in the present study, exposing (100) and (010) surfaces. The adlayer consists of Porph at intermediate coverage. The entire sample consists of 61 cellulose chains and 96 Porph molecules. White dots, H; black, C; blue, N; red, O. The black arrows indicate the elongation axis of the CNF.

A CNF sample has been prepared by cutting the bulk by planes perpendicular to the **a** and **b** directions, thus exposing pairs of (100) and (010) facets. Despite the relatively high γ_s_ of the (010) surface, a long equilibration of the neat fiber at *T* = 300 K did not change the shape of the sample, consisting of 61 chains. Even the progressive heating to *T* = 360 K and annealing back to *T* = 300 K, following the annealing schedule used for the planar interfaces, did not change the structure of the neat nanofiber, apart from a very limited rounding of the corners (see Figure 13). Isotropic addition of molecules results in samples in which are recognizable most of the features described in the previous subsections. A regular arrangement on the (100) facets is observed for Porph at all coverages, and for TPP and TMPyP at submonolayer coverage, while 3D clusters form starting from monolayer coverage in all the other cases, and, in particular, on the (010) facets. Also, the addition of water to the TMPyP samples produces effects similar to those described in the case of planar adlayers.

In the Porph case, the edges parallel to the fiber axis seem to act as potential energy barriers, since they are relatively deprived of admolecules, thus limiting the transfer of molecules between adjacent facets. Because of low mobility, the morphology of TPP and TMPyP adlayers beyond the low coverage regime is determined by the random addition process. Locally, the growth can be recognized as Stranski–Krastanov.

**Figure 14:**
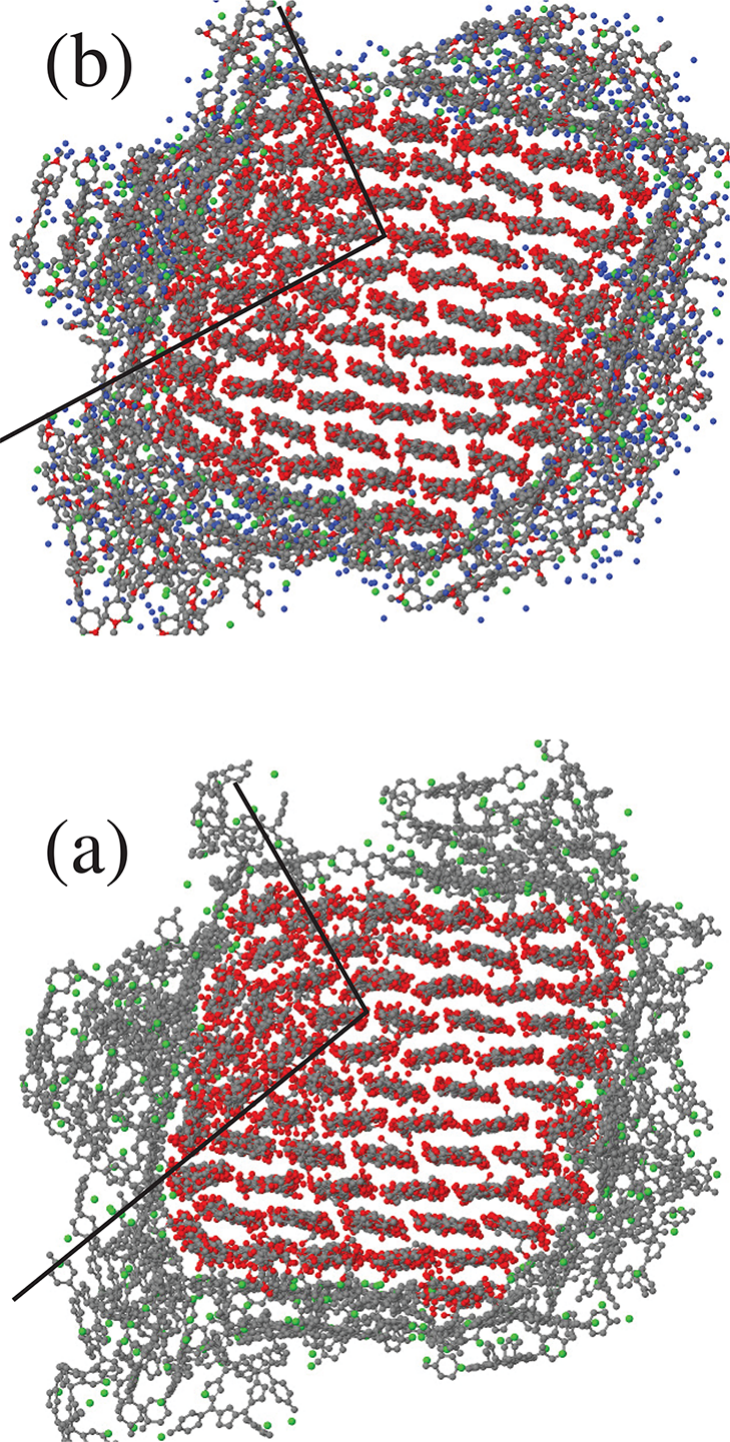
Cross sectional view of the CNF described in the text covered by 80 TMPyP molecules, dry in panel a, and following the addition of 1000 water molecules in panel b. The overall similarity of the two structures is due to the fact that the wet sample was started by adding water to an early dry configuration, and both samples have been simulated for a further 100 ns at *T* = 300 K. The black lines in both panels highlight the approximate contour of a crystalline grain misoriented with the original structure. Carbon and oxygen atoms in cellulose are drawn black and red, respectively. Carbon and nitrogen atoms in TMPyP are drawn black. Cl^–^ ions are green. Water oxygens in panel b are drawn blue. Hydrogens are not shown.

The interaction with porphyrin admolecules perturbs the cellulose structure more than in the extended slab cases, especially at the edges and on the (010) facets. Because of its ionic character, TMPyP interacts more strongly than Porph and TPP, and at high coverage it drives the nucleation of a different crystal grain in the cellulose fiber (see Figure 14). The same effect is observed both in dry samples and in wet samples, prepared by adding 1000 water molecules to the dry samples, as seen by comparing panels a and b of Figure 14. Because of the lubrication property of water, the structural evolution of cellulose is faster in the wet sample than in the dry one.

## IV Summary and Conclusions

Extensive molecular dynamics simulations based on a rigid-ion empirical force field show that low-index cellulose crystal surfaces provide a thermally stable and weakly perturbing substrate for the deposition of non-hydrogen-bonded organic species, exemplified by three porphyrin species. Simulations have been carried out for planar cellulose slabs in 2D periodic boundary conditions, and for one model nanofiber of ∼4.5 nm diameter, exposing (100), (010), and (110) surfaces and facets.

In the present study, the growth of Porph, TPP, and TMPyP adlayers on cellulose surfaces is simulated as occurring by molecular vapor deposition. At low coverage (θ < 0.6), the disk shaped porphyrin molecules arrange themselves flat on the (100) and (110) surfaces and form compact 2D islands displaying geometric motifs familiar from experimental measurements of physisorbed epitaxial porphyrin layers on graphite and smooth surfaces of noble metals and organic crystals. The structure of the islands is compatible with their further growth in either a square or hexagonal 2D lattice. This low selectivity of surface structures is compatible with the extensive polymorphysm of TPP overlayers grown on flat metal or crystal salt substrates (see, for instance, ref (26)).

The surface mobility of Porph on the compact (100) and (110) surfaces is high, allowing the system to reach a near equilibrium ordered configuration. Ordering persists up and beyond the monolayer, giving a solid-like overlayer made of slightly leaning columns growing on the cellulose surface. In all other cases, including Porph/(010), low surface mobility and perhaps the combination of geometric mismatch with the periodic boundary conditions and microscopic simulation times prevent the formation of a fully ordered extended monolayer. A second layer starts to form before θ = 1 coverage, and growth continues in the form of 3D clusters, corresponding to the Stranki–Krastanov model.

The formation of ordered structures on smooth cellulose surfaces up to near monolayer coverage is good news for applications of (usually metal-substituted) porphyrins in catalysis, bactericidal and virucidal surfaces, and electronic and optoelectronic devices. The growth by 3D clusters beyond the first monolayer is certainly less favorable for applications, but the results on higher mobility and regularity in ionic porphyrin overlayers contaminated by water point to possible strategies to enhance the formation of thick ordered adlayers, from which water might be removed afterward.

The structural and dynamical properties described in our paper could be verified by STM measurements, by advanced electron microscopy imaging, and by inelastic scattering of neutral atomic (He) beams. The pillars formed by porphine on cellulose (100) could support organ-pipe vibrational excitations, similar to those observed in other pillar structures and revealed by inelastic He scattering.^56^ The same method could probe the presence of Levy flights, and superlubricity could be investigated by standard tribology methods.

In many ways, the role and the effect of the cellulose substrate do not seem to be different from what could be expected from a plastic surface, confirming that plastics could be replaced by cellulose in many applications, including laboratory equipment. Moreover, and more importantly, the inherent nanocrystallinity of cellulose might represent an advantage for some applications, for instance, in terms of reproducibility. The nanocrystalline facets of CNFs in combination with porphyrins, moreover, could display a variety of epitaxial effects at near monolayer coverage, which could open the way to thermoresponsive adlayers for sensing and for storage nanodevices.

## Supporting Information Available

Topographic view of a cellulose–porphine interface, plots of cellulose surface energy and entropy as a function of temperature, adsorption energy and free energy of porphine on cellulose, square displacement of porphine on cellulose as a function of time, rotational time autocorrelation function, orientation of porphine on cellulose, anisotropic dispacement of porphine on Iβ cellulose (010), snapshots of high-coverage porphine on cellulose, snapshot of TMPyP adlayers, height–height surface correlation function, snapshot of water droplets on cellulose, and dry and wet TMPyP adlayers on cellulose following thermal annealing (PDF)
