# Supplementary material for: Morphology of Nanometric Overlayers Made of Porphyrin-Type Molecules Physisorbed on Cellulose Iβ Crystals and Nanocrystals
Source: J Phys Chem B. 2021 Oct 12;125(41):11432–43. doi: 10.1021/acs.jpcb.1c07261 (PMC8543442; doi:10.1021/acs.jpcb.1c07261)
Supplement: Supplementary file 1 — jp1c07261_si_001.pdf [file jp1c07261_si_001.pdf]

Supporting Information on:

Morphology of Nanometric Overlayers Made of Porphyrin-type Molecules Physisorbed on Cellulose Iβ Crystals and Nanocrystals

Agata Fularz,[1,2] James H. Rice,*[1,2] and Pietro Ballone[1,2]

(1) School of Physics, University College Dublin, Dublin 4, Ireland and

(2) Conway Institute for Biomolecular and Biomedical Research, University College Dublin, Dublin 4, Ireland

∗ *Corresponding Author: James H. Rice James.Rice@ucd.ie*

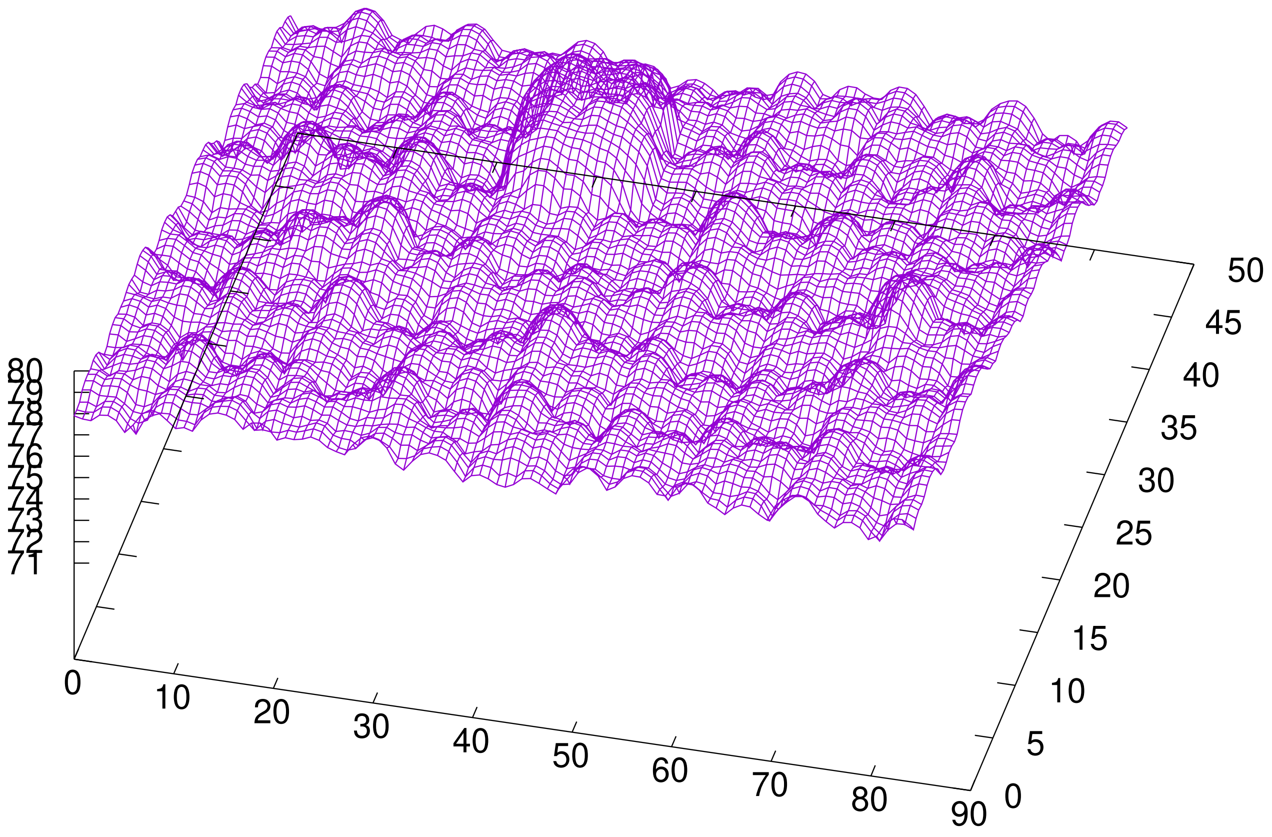

FIG. S1: Topography of the (100) cellulose surface determined by the computational AFM method described in the main text. The blob on the surface corresponds to an adsorbed porphine. All scales are in Å .

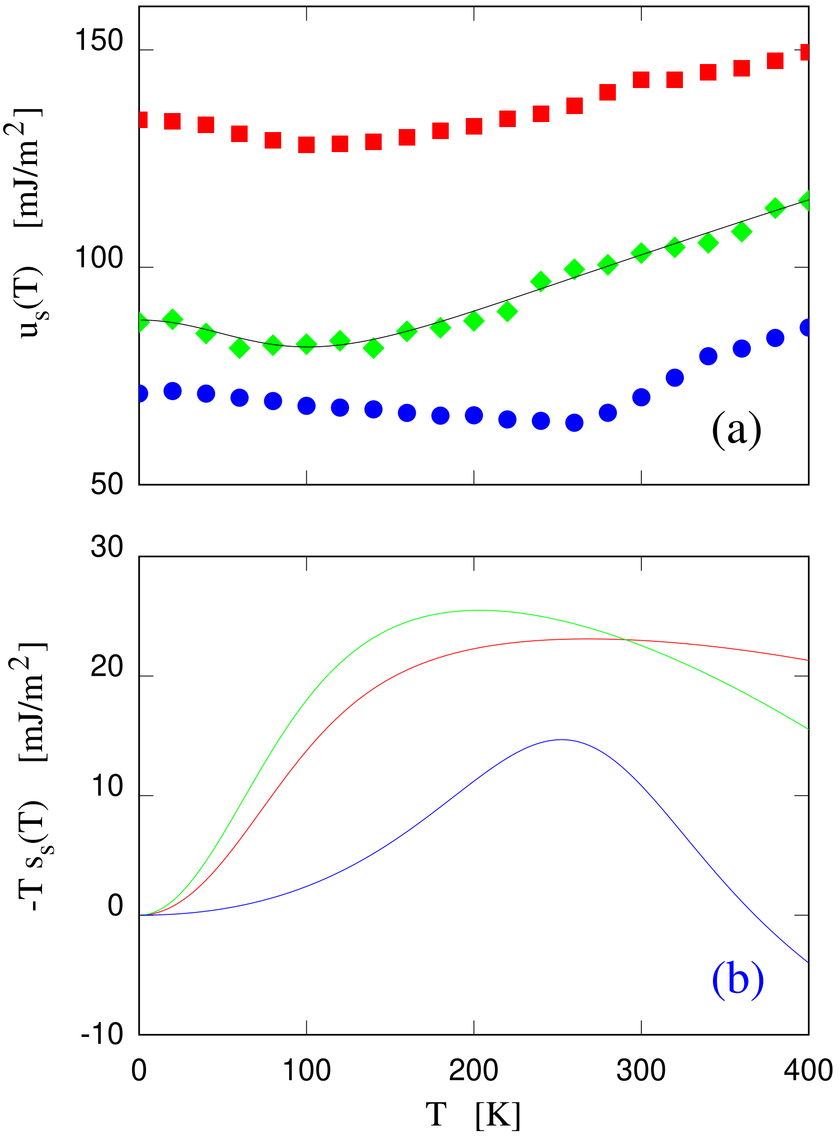

FIG. S2: Temperature dependence of: (a) the surface energy u_s and (b) the surface entropy s_s of cellulose Iβ crystal surfaces. Symbols and lines of the same color refer to the same surface. Blue: (100); green: (110); red: (010).

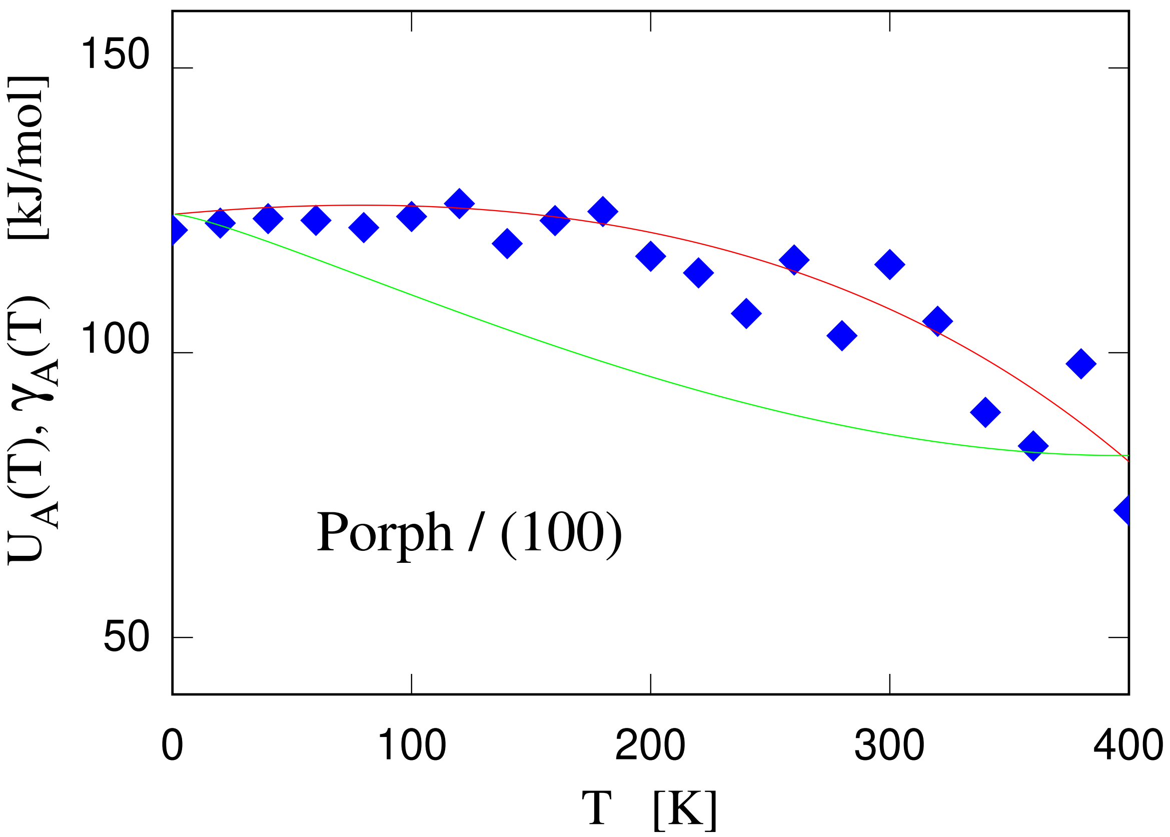

FIG. S3: Adsorption energy (U_A, blue diamonds) and free energy (γ_A, green line) of one Porph molecule on the (100) surface of cellulose. The red line is the interpolation of the computations data (blue diamonds) used to determine the entropy term in γ_A. The scattering of data at $T \geq 300$ K is due to the fact that the adsorption energy is a small difference of two large, fluctuating potential energies, computed for the clean cellulose slab and for the slab with one adsorbed Porph. The definition of U_A is such that positive values correspond to an attractive interaction between surface and adsorbate.

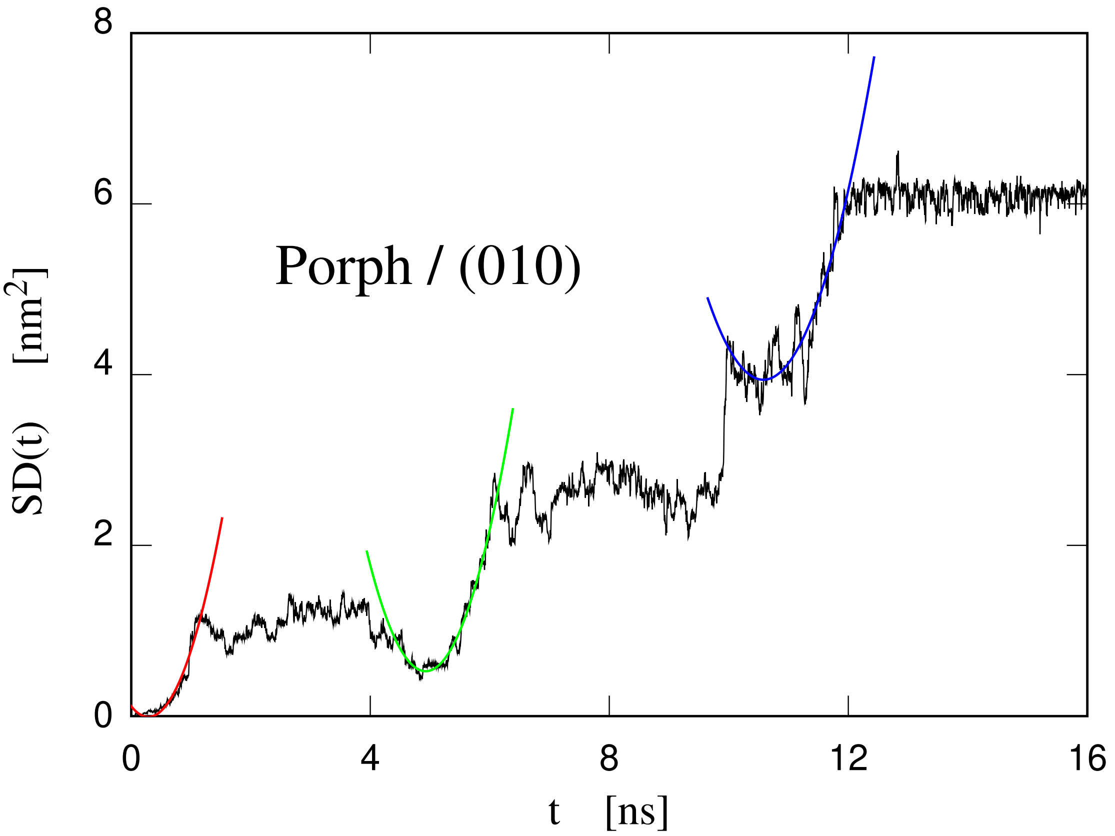

FIG. S4: Black line: square displacement of the Porph centre of mass as a function of time: $SD(t) = \langle| \mathbf{R}_{CM}(t + t_0) - \mathbf{R}_{CM}(t_0) |^2\rangle_{t_0}$. The curve has been computed along a 20 ns segment of the trajectory taken from the third quarter of a 200 ns trajectory, shifting the origin of time and coordinates accordingly. The color curves are quadratic interpolations of subintervals of $SD(t)$, selected on the basis of visual impression. The parabolic intervals correspond to displacements comparable or larger than the Porph diameter, and point to nearly rectilinear displacements only weakly perturbed by the interaction with the substrate.

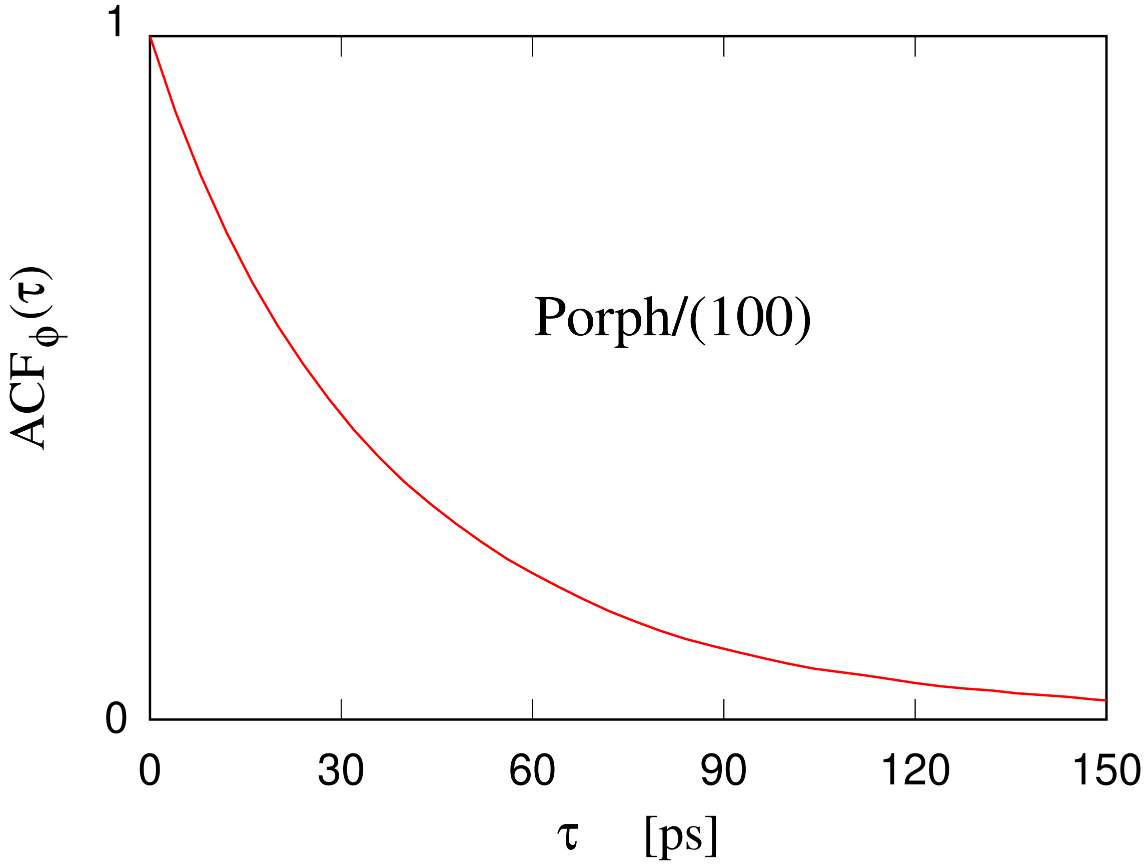

FIG. S5: Time autocorrelation function of the molecular orientation on the cellulose surface, defined through a unit vector joining the two NH groups of Porph and the cellulose chains axis (i.e., the z axis). $ACF_\phi(\tau)$ characterises the rotational diffusion of the ad-molecule. The function in the figure refers to a single Porph molecule on the surface of the (100) slab.

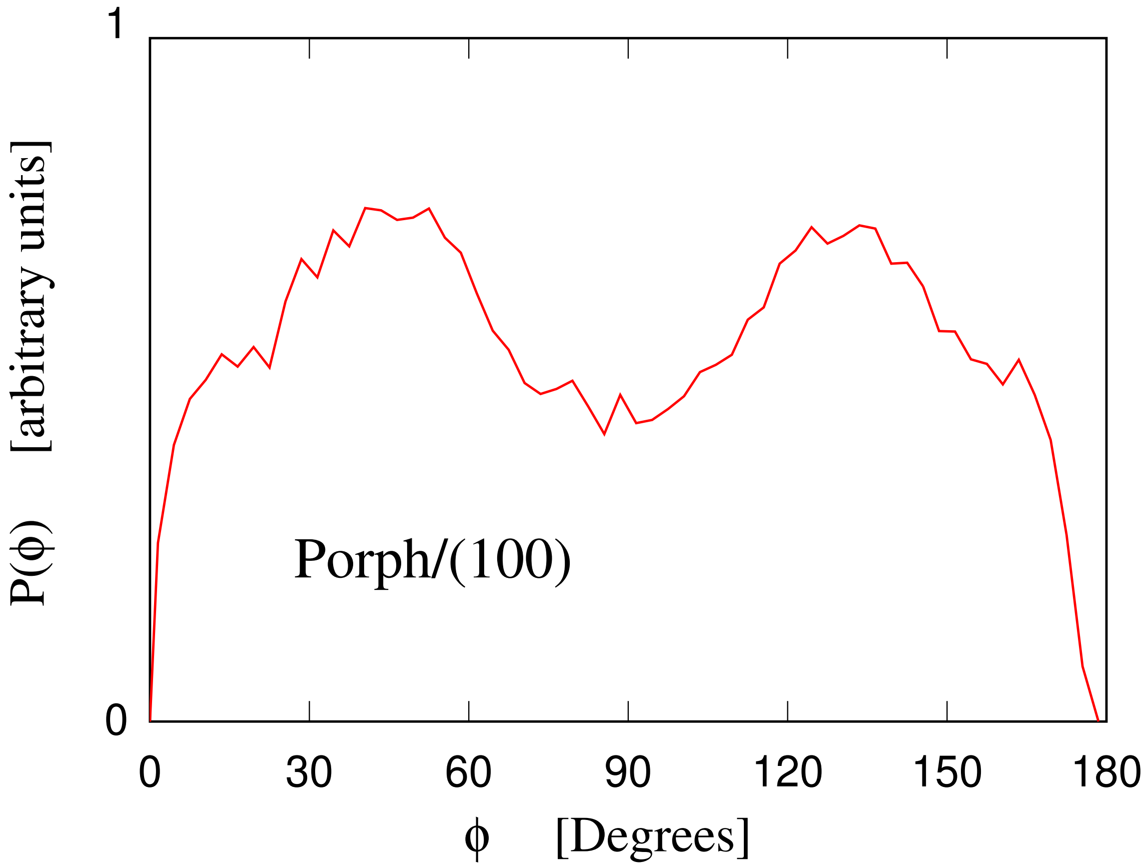

FIG. S6: Probability distribution of the angle ϕ between the unit vector joining the two NH groups of Porph and the cellulose chains axis (i.e., the z axis). The area under the curve is normalised to 100. The peaks at $\sim$ 45 and $\sim$ 135 degrees account also for those at 225 and 315 degrees. The histogram refers to a single Porph molecule on the surface of the (100) slab.

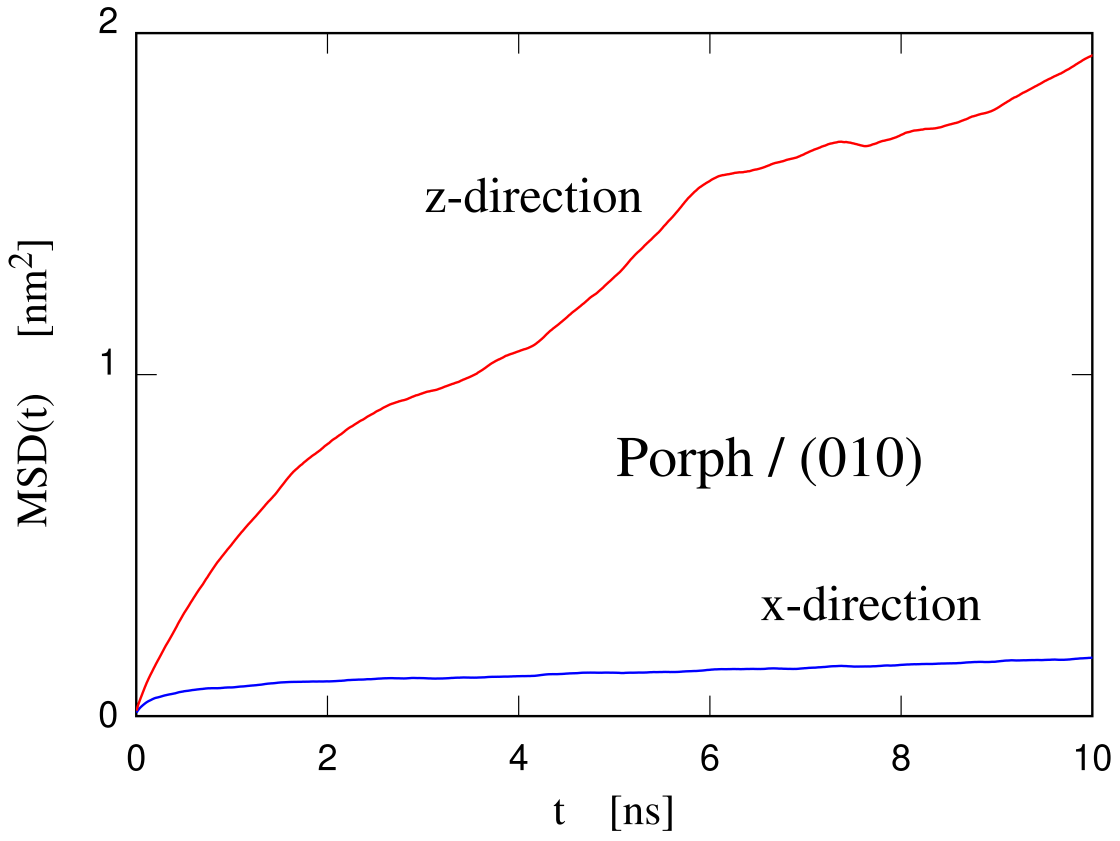

FIG. S7: Mean square displacement of a single porphine molecule on the (010) surface of cellulose Iβ. Blue line: along the x direction; red line: along the z direction. The corresponding MSD along y is nearly time independent, and too small ($3\ 10^{-2}$ nm^2) to be plotted on the scale of the figure. The 1D diffusion constant estimated along z is $D_x = 1 \pm 0.2\ 10^{-6}$ cm^2/s. The corresponding value along x is $D_y = 5 \pm 2\ 10^{-8}$.

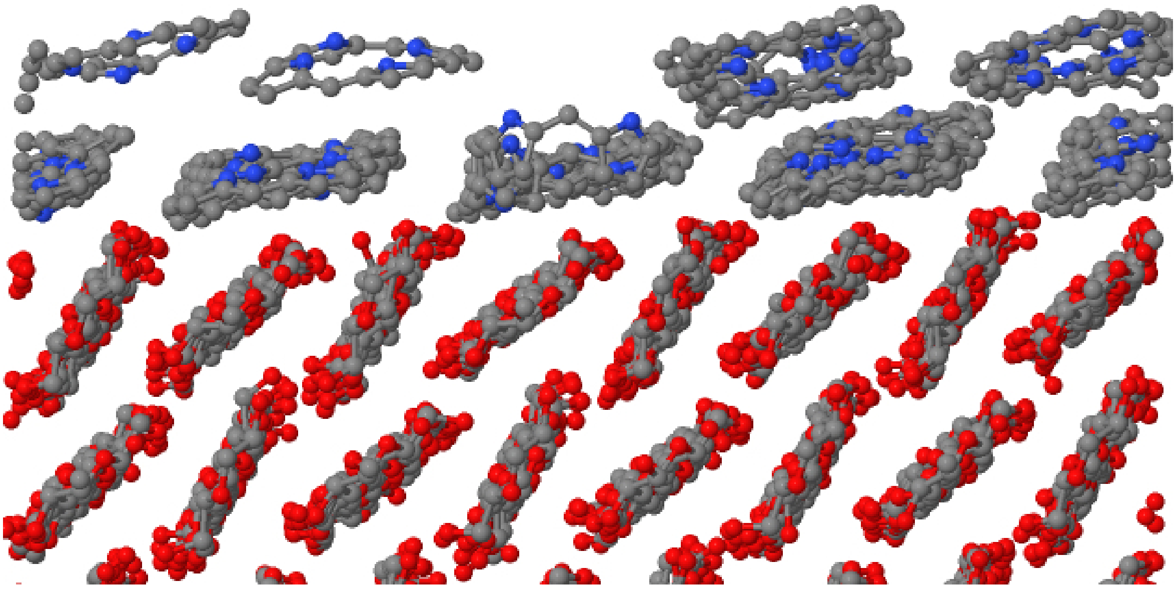

FIG. S8: Snapshot of portion of the Porph adlayer on (110) surface at coverage corresponding to 40 Porph molecules on the (110) slab described in the main text, whose surface area is $A = 3614$ Å ^{2}. Black dots: C; blue: N; red: O. Hydrogen atoms not shown.

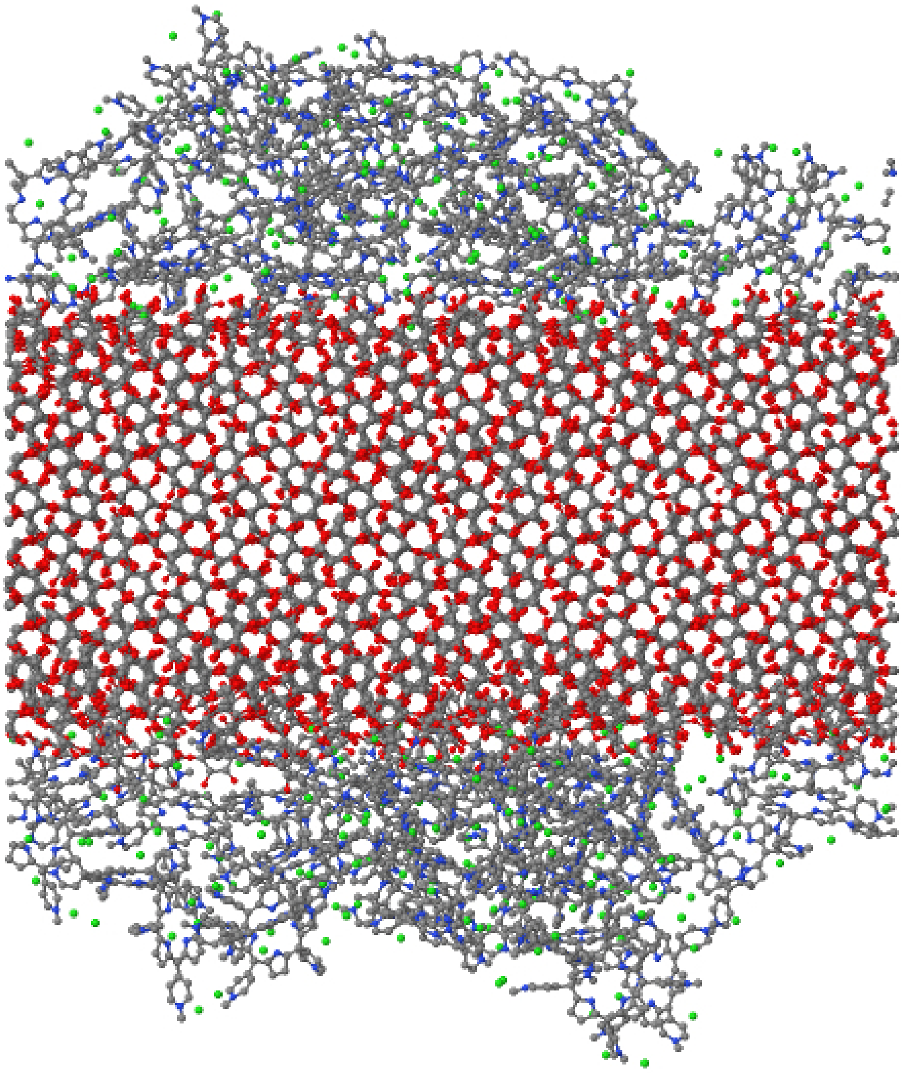

FIG. S9: Snapshot of TMPyP adlayer on the (010) surface (40 molecules per surface of 3988 Å ^{2}) displaying growth by disordered 3D clusters. Black dots: C; blue: N; red: O, green: Cl$^-$. Hydrogen atoms not shown.

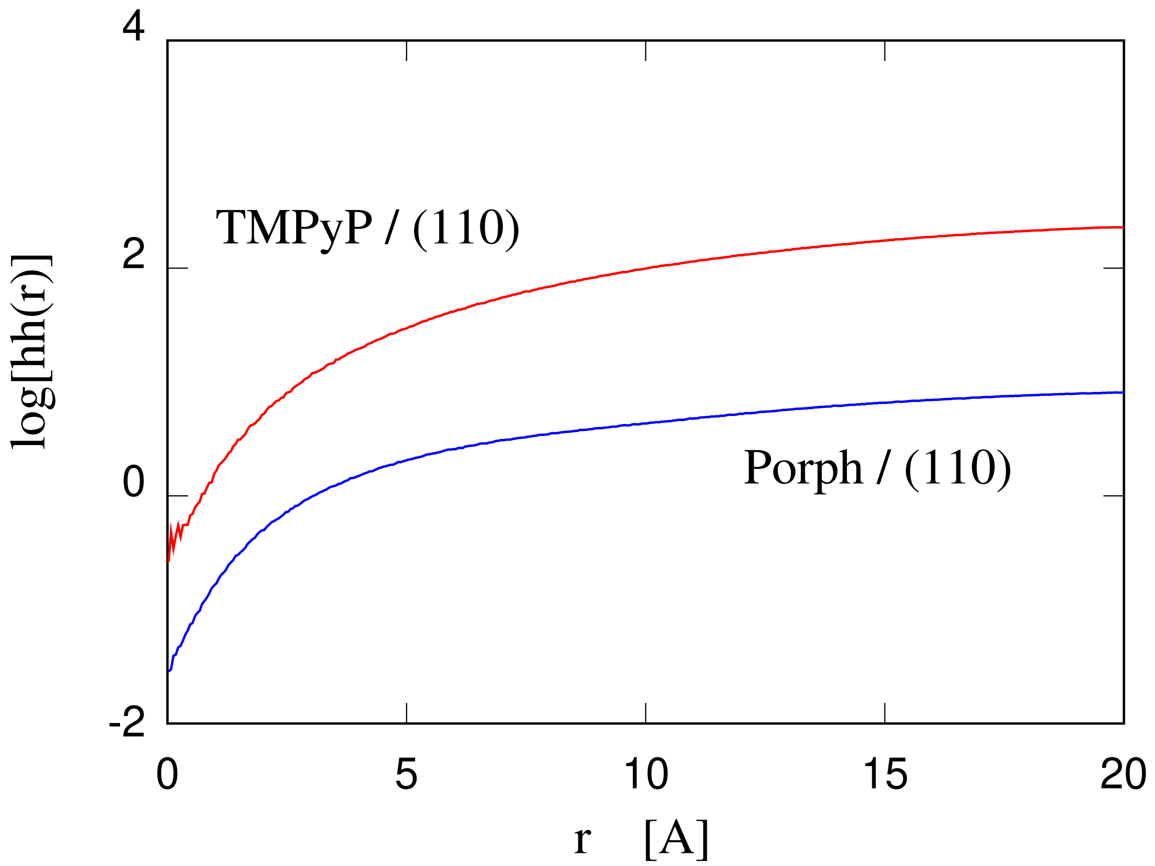

FIG. S10: Semi-logarithm plot of the height-height correlation function $hh(r)$ (see the definition in the main text) for Porph and TMPyP adlayers on the (110) cellulose surface. The coverage corresponds to 40 molecules on the (110) slab defined in the main text, whose surface area is $A = 3614$ Å ^{2}. The range of r in the figure is slightly less than half the shortest periodic distance on the surface, too short to characterise the adlayer surface as smooth or rough. $hh(r)$ is measured in Å ^{2}. The plotted range, relatively unaffected by periodic boundary conditions, is too narrow to reach an unambiguous conclusion on the smoothness/roughness of the surfaces.

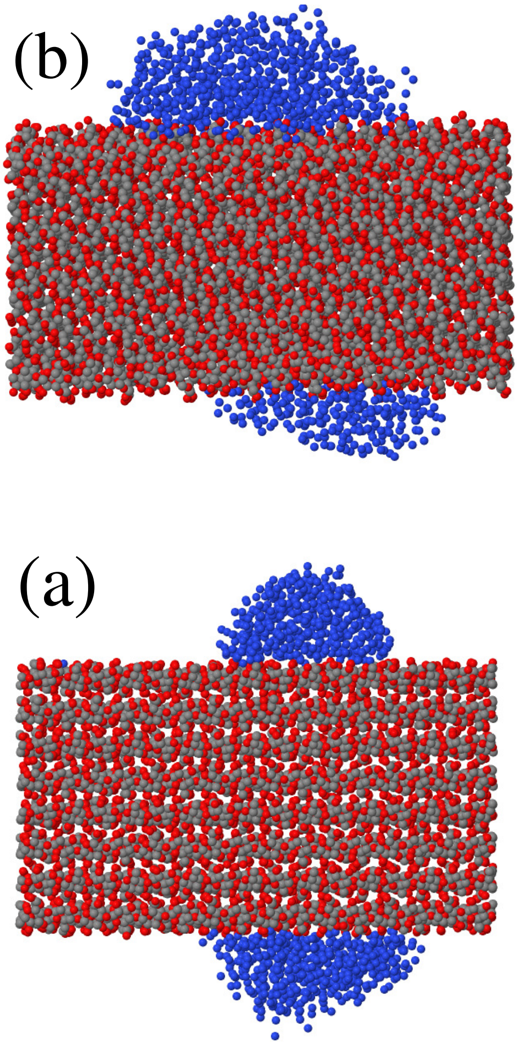

FIG. S11: Water droplet resulting from the condensation of a random distribution of 1000 water molecules in the empty regions of the simulation box for: (a) the (periodically extended) slab exposing two (110) surfaces; (b) the crystal nanofibre described in the main text. Carbon and oxygen atoms of cellulose are painted black and red, respectively. Water oxygen is painted blue. Hydrogen atoms are not shown.

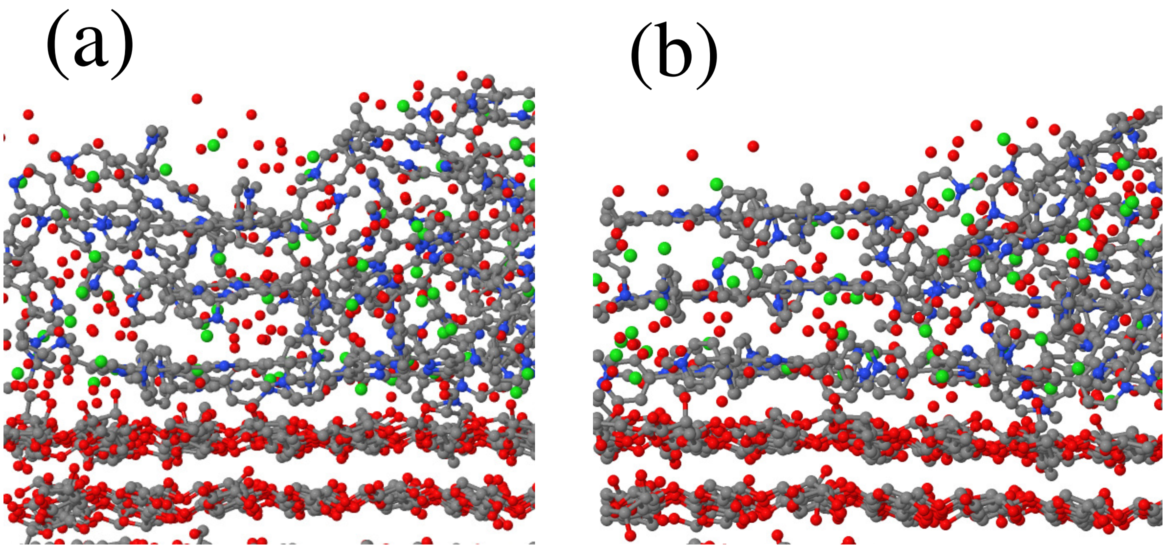

FIG. S12: Snapshot of the wet TMPyP / (100) sample (a) before and (b) after the 120 ns annealing cycle described in the main text. Black dots: C; blue: N; red: O, green: Cl^-. Hydrogen atoms not shown.
